# Determinants of correlated expression of transcription factors and their target genes

**DOI:** 10.1093/nar/gkaa927

**Published:** 2020-10-26

**Authors:** Adam B Zaborowski, Dirk Walther

**Affiliations:** Max Planck Institute of Molecular Plant Physiology, Am Mühlenberg 1, 14476 Potsdam-Golm, Germany; Max Planck Institute of Molecular Plant Physiology, Am Mühlenberg 1, 14476 Potsdam-Golm, Germany

## Abstract

While transcription factors (TFs) are known to regulate the expression of their target genes (TGs), only a weak correlation of expression between TFs and their TGs has generally been observed. As lack of correlation could be caused by additional layers of regulation, the overall correlation distribution may hide the presence of a subset of regulatory TF–TG pairs with tight expression coupling. Using reported regulatory pairs in the plant *Arabidopsis thaliana* along with comprehensive gene expression information and testing a wide array of molecular features, we aimed to discern the molecular determinants of high expression correlation of TFs and their TGs. TF-family assignment, stress-response process involvement, short genomic distances of the TF-binding sites to the transcription start site of their TGs, few required protein-protein-interaction connections to establish physical interactions between the TF and polymerase-II, unambiguous TF-binding motifs, increased numbers of miRNA target-sites in TF-mRNAs, and a young evolutionary age of TGs were found particularly indicative of high TF–TG correlation. The modulating roles of post-transcriptional, post-translational processes, and epigenetic factors have been characterized as well. Our study reveals that regulatory pairs with high expression coupling are associated with specific molecular determinants.

## INTRODUCTION

The regulation of gene expression is one of the most essential processes in the control of cellular functions and development. Largely driven by technological advances, but also because of the central biological importance of transcriptional regulation, transcriptional information has become the main type of information collected in many experiments, which has led to a widespread availability of comprehensive publicly available datasets generated for many cell-types, tissues, and organisms exposed to many different experimental conditions.

In the analysis of the generated expression data, the inference of gene regulatory networks (GRNs) is a central goal, i.e. discerning the interplay between transcription factors (TFs) and the genes they regulate, their target genes (TGs), based on gene expression information. Many computational approaches to predict regulatory connections have been developed (for review, see (1–4)) that apply a wide range of mathematical and computational methods (5,6), combinations thereof (7), as well as approaches that include prior biological knowledge about genes and their interactions at the level of proteins and mediated via metabolites (8,9). However, the results obtained so far proved far from perfect, neither for eukaryotic organisms, such as *Saccharomyces cerevisiae* or *Arabidopsis thaliana* (7,10), where gene expression regulation is thought to be under multi-layer control and, therefore, challenging to predict, nor for prokaryotic organisms such as *Escherichia coli* (7,11), where regulatory processes are believed to be less complex. The unsatisfactory performance could be caused by algorithmic limitations of the statistical inference methods (12), or, more principally, by the absence of a direct association of expression between regulating, TFs, and regulated genes, TGs. When attempting to infer GRNs from gene expression data, we assume that changes of the expression of genes encoding TFs are followed by expression changes of the TGs they regulate. Hence, a correlated gene expression profile, temporal or across different conditions, should logically follow, which, in reverse, can be exploited to infer regulatory interactions from expression data. Indeed, in the prokaryote *E. coli*, experimentally verified regulatory gene pairs show higher gene expression correlation levels between TFs and their TGs than between TFs and non-TGs (13). However, when probed in eukaryotes, where additional layers of gene expression regulation have evolved, the correlation of expression between TFs and their known TGs proved to be only slightly higher than the expression correlation between TFs and randomly selected genes (7).

As TFs exert their function as proteins and not as transcripts, their function can be modulated by many factors, which could act independently of the expression of TFs, such as the rate of translation and protein degradation, regulation by post-translational modifications (PTMs), and interactions with other proteins required to initiate transcription (14). It can be also modified by properties and functional states of the promoter sequence of the respective TGs, e.g. the openness of chromatin, epigenetic markers, or presence of other TFs, which bind to this DNA region (15,16). Those processes in-between the expression of TFs and their resulting activity, leading to the activation or repression of a TG, can heavily influence on the functional significance of expression correlation levels between TFs and their TGs, even uncoupling correlation and functional relevance completely, and for principal reasons. This may mean that for the principal reasons described above, no consistently strong expression-based signal that separates true from false TF–TG pairs is discernible in higher organisms, such that inferring GRNs from expression data alone would essentially be a futile exercise. However, it is also possible that the small, but nonetheless discernible shift towards increased correlation values among true vs. false pairs is associated with a subset of TF–TG pairs exhibiting relatively tight correlations, which can furthermore be associated with specific gene-related or genome structural properties.

Here, we address the question whether for experimentally verified TF–TG pairs, those that exhibit high expression correlation are characterized by specific gene-related or genome structural properties that are different from those TF–TG pairs that appear uncorrelated. Alternatively, our study may reveal that no such set differences exist and that the small, but detectable shift to increased correlation among true versus false regulatory pairs is merely a general signal that is weakened by the many influencing factors discussed above. Furthermore, our study goal also allows us to estimate the extent of direct transcriptional regulation and to assess the importance of additional layers of gene expression regulation that may obscure expression correlation.

We performed our analysis in the well characterized plant *A. thaliana*, for which a thoroughly curated genome, broad expression information obtained for different developmental stages, stress conditions and other experiments, and, critically for this study, a dataset of candidate true TF–TG pairs is available. The recently published (Plant Cistrome Database (PCD), (17)) contains information about experimentally identified TF–genomic–DNA binding events and associated binding-sites (TFBSs) for 386 selected TFs of *A. thaliana*, and thus, knowledge about TFs and their likely TGs. Therefore, it provides the basis to systematically probe the expression correlation of TFs and their candidate TGs in *A. thaliana*, with the goal to determine whether there are indeed characteristic molecular properties for expression-correlated versus uncorrelated pairs, respectively. As for molecular properties, we considered a wide array of genomic (e.g. sequence composition, positional information of TFBSs relative to the transcription start site (TSS) of genes), genome structural (e.g. distances between genes), higher levels of gene expression regulation (e.g. PTMs, protein–protein interactions, chromatin state), and evolutionary parameters (‘age’ of genes). Each considered property was added based on a specific rationale potentially linking it to gene expression regulation. For example, it is possible that TF-transcripts and -proteins are always present at constant levels in cells, but the TF-proteins are activated and deactivated via phosphorylation status changes by kinases and phosphatases acting on them. In that case, no expression correlation signal between those TFs and their TGs is to be expected. As a surrogate to address this question, we tested the number of potential phosphorylation sites on TFs to serve as a criterion that may distinguish correlated (few phosphorylation sites) from uncorrelated (many phosphorylation sites) TF–TG pairs. Similarly, genes involved in essential housekeeping functions may always be expressed at relatively constant levels and, therefore, show reduced levels of correlation with TFs, while genes linked to response-to-environment processes may exhibit a larger differential dynamic range and hence increased correlation. As the former may be evolutionarily older with response genes evolving later in evolution, we tested for the role of evolutionary age of genes. As it has been shown before that genomic features such as length of a gene or distance to the next upstream gene is correlated with differential gene expression behavior (18), we tested for their relevance with regard to pairwise correlations. This study was also motivated by our initial observations that unexpectedly, particular TF-families are significantly enriched in the set of correlated TF–TG pairs. And as the assignment of TFs to belong to a particular family must have molecular correlates or may reflect specific modes of gene expression regulation, we wished to discern those.

To identify informative molecular determinants and hence learn about regulatory mechanisms, we performed univariate statistical tests on the set of selected candidate features deemed relevant in the modulation of gene expression (135 in total) and trained a machine learning (ML) model (Random Forest Classifier) to discern multivariate effects as well as to unravel interactions between the selected features, and to gauge the predictability of correlation given prior information. The ML-model proved indeed capable of distinguishing highly correlated from non-correlated TF–TG pairs. By discussing the discerned informative features with regard to possible mechanistic relevance, our study sheds light on the orchestration of transcriptional regulation in eukaryotes as represented by the plant *A. thaliana*.

## MATERIALS AND METHODS

### Transcription factor (TF)–target gene (TG) pairs, DAP-seq data

The set of true interactions of transcription factors (TFs) and their target genes (TGs) was defined as the set of physical interactions and associated genomic positions as reported in the PCD (17). This database provides information on TFs binding to genomic DNA obtained from DNA affinity purification assays combined with sequencing (DAP-seq) for 386 *A. thaliana* TFs. TF-binding sites (TFBS) were obtained by scanning the reported peak regions from DAP-seq experiments, with binding motifs provided as position weight matrices (PWM) for every TF in the PCD reported in O’Malley *et al.* (17) and using the ‘TFBSTools’ package available from the Bioconductor repository, with the background nucleotide composition obtained for each TFs from PCD and with a minimal score threshold set to 80%, which represents the quantile between the minimal and the maximal possible value from the respective PWM (19). Genes were considered targets of a TF, if an associated DAP-seq peak with a correspondingly present TFBS was found located in the respective gene promoter region. Gene promoters were defined as the genomic interval of –500 bp to –1 bp from the transcription start site (TSS) of a gene, consistent with the reported effective promoter length in *A. thaliana* (20). The location of respective TSSs was taken as the annotated transcription start site according to the TAIR-10 (21) annotation file. Genes without annotated 5′UTR sequences (7679 genes, including 59 TFs with 5′UTR sequence length of zero) have been removed from the set of considered TFs and TGs to avoid false promoter and TSS annotations. In total, the dataset comprised 280 655 TF–TG pairs, created between 290 TFs and 15 852 TGs with respectively available expression information (presence on ATH1-chip, see below). Distances between TFBS and TSS were computed with bedtools’ ‘closest’ command (22), which determines the respective closest distance to a TSS of neighboring genes, regardless of direction.

### Expression data

As the primary source of expression information, data were obtained from a set of 5296 hybridizations on Affymetrix ATH1 expression microarrays, as available from NASC database (http://arabidopsis.info/affy). Natural logarithm of all expression values was calculated for the 15 852 genes that are part of the DAP-seq set, have annotated 5′UTR, and are present on the expression microarrays. Mapping of ATH1-mircoarray identifiers to AGI-identifiers was required to be unique. Expression data were further normalized by quantile normalization using the ‘normalize.quantile’ routine from the ‘preprocessCore’ R package (23).

Expression data using the RNA-seq technique was obtained from TravaDB (24) for 158 biological samples. Reads per kilobase per million mapped reads (RPKM) was obtained for each of 33 323 genes for all 158 samples, and log-transformed (log(RPKM + 1) to render them normally distributed.

### Correlation of expression and active TFs selection

Pairwise Pearson correlation coefficients were calculated between the expression values of all 5296 microarray hybridizations values associated with the 290 TFs present in the PCD and the 15 852 genes present in the expression microarray dataset. From the matrix of correlation coefficients, we selected the correlation coefficients of the 280 655 pairs, which were reported in the PCD as candidate regulatory pairs, i.e. pairs of TFs and TGs with the respective TFBS present in their promoter regions and with a proper annotation of the 5′UTR. The set of those 280 655 TF–TG pairs was taken as the set of true regulatory pairs. For comparison, correlation coefficients for TFs and non-TGs pairs were computed for each TF and randomly selected genes, which were not assigned as TGs in the PCD for the selected TF and were present in the expression dataset. The number of non-TG pairs for each TF was chosen to match the number of true TGs pairs for this TF.

**Figure 1.**
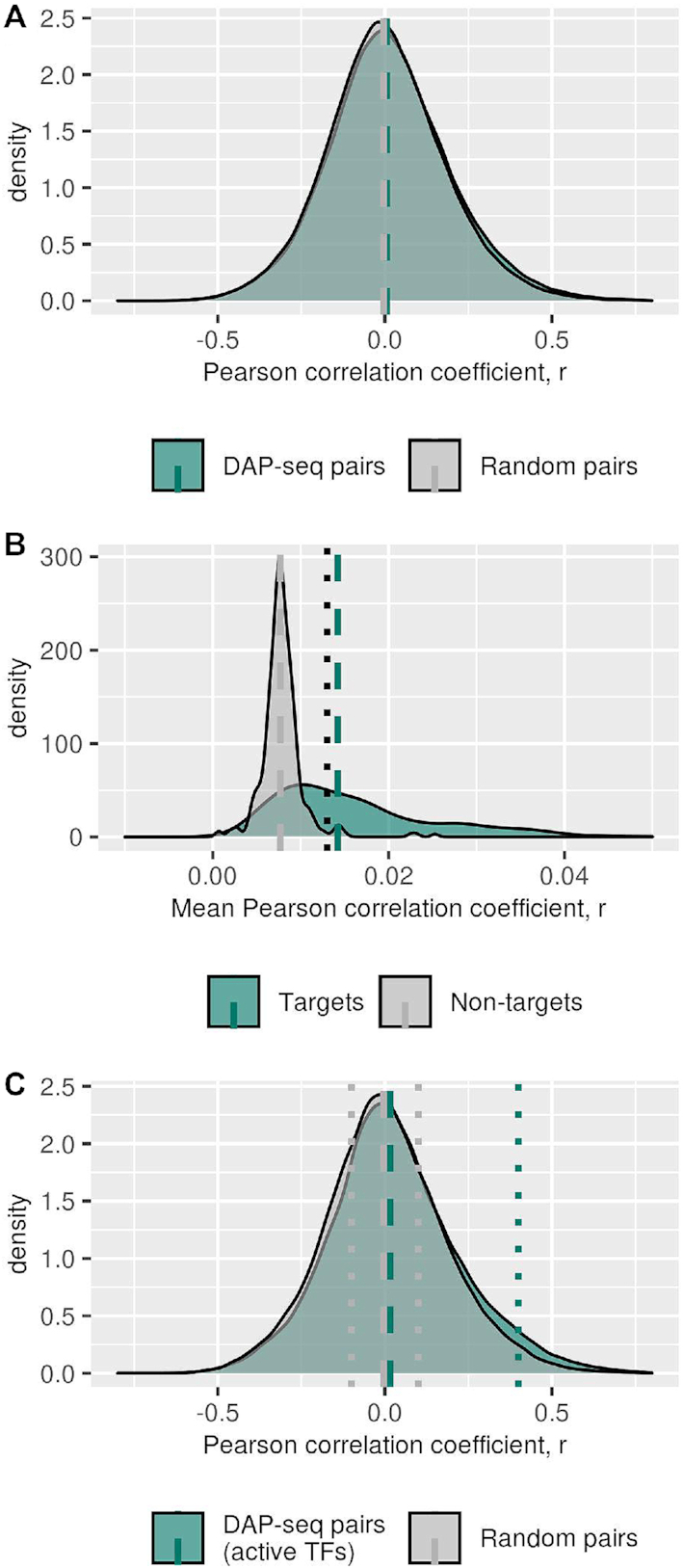
Distribution of Pearson correlation coefficients of TF–TG pair expression. (**A**) Expression correlation between transcription factors (TFs) and their target genes (TGs) (green) and random non-target genes (non-TGs) (grey) for all TFs. Dashed vertical lines represent the median Pearson correlation coefficient for regulatory pairs obtained from DAP-seq (green) and pairs with random genes (grey), median values 0.006 and -0.005 respectively (*P*-value < 2.2E–16 Wilcoxon–Mann–Whitney test; effect size = 0.07). (**B**) Frequency distribution of pairwise correlation coefficient of genes identified as TGs of TF (green) and randomly selected non-TGs (gray). For every TF, the mean pairwise correlation of gene expression values for all its TGs was computed. For comparison, size-matched sets of random genes were drawn and their respective mean pairwise correlation coefficient computed. The histograms were obtained over all such mean values. TFs with associated TG-sets that exhibit pairwise correlation above a correlation level of *r* > 0.013 (dotted line, *r*-value greater than 97.5th percentile of the randomly selected non-TGs) were defined ‘active’. Dashed lines represent median values of expression correlation for TGs (green), 0.014 and non-TGs (gray), 0.008 (*P*-value < 2.2E–16 Wilcoxon–Mann–Whitney test). Notably, the mode of distribution of the random pairwise correlation coefficients is not on or near-zero correlation. This may result from a residual global correlation of all genes across all hybridization samples resulting from imperfect normalization or even a true global residual correlation. (**C**) Expression correlation between transcription factors (TFs) and their target genes (TGs) (green) and random non-target genes (non-TGs) (gray) for set of selected active TFs. TFs were defined as ‘active’ if their respective TGs exhibit significant pairwise expression correlation (Figure 1B). Dashed vertical lines represent the mean Pearson correlation coefficient, r, for regulatory pairs obtained from DAP-seq (green) and pairs with random genes (gray), median values 0.018 and –0.010 respectively (*P*-value < 2.2E–16 Wilcoxon–Mann–Whitney test; effect size = 0.16). Dotted vertical lines represent the threshold for the selection of correlated pairs (black), *r* > 0.4 and gray dotted lines represents uncorrelated pairs selection with –0.1 < *r* < +0.1 (gray).

For every TF, Pearson correlation coefficients of the expression of all its TGs (all-against-all) were calculated, along with the correlation of expression of the same number of pairs of non-TGs for this TF. If the number of TGs of the TF was larger than 3000, we used a subset of 3000 TGs randomly selected from the set of all TGs associated with the selected TF. Of the set of 290 TFs, TFs were designated ‘active’, if the average of the pairwise correlation of the associated TGs was above a threshold value of 0.013 (Figure 1B), leaving 157 active TFs for analysis. This threshold was determined as the 97.5th percentile of random non-TGs pair averages over all TFs. From the set of all 280 655 pairs, 104 369 pairs with active TFs were selected. Pearson correlation coefficients were calculated with the function ‘cor’ from ‘stats’ R-package (25).

To compare results obtained from two different expression profiling techniques, microarrays and RNA-seq, we assessed the concordance of TF–TG correlation by calculating the correlation between TF–TG-pair-correlation values obtained for both techniques. A subset of 15 838 TGs and 280 TFs was present in both sets. For the associated set of 268 090 TF–TG pairs, we calculated their Pearson correlation coefficients in the two expression datasets. With the Pearson correlation coefficient assigned for each pair calculated from both expression datasets, we computed the Pearson correlation between those coefficients for all TF–TG pairs.

### TF-family assignments and enrichment analysis

TF-family assignments of all TFs were obtained from PlnTFDB (26). The 157 active TFs represented in the PCD were found to belong to 24 families (Table 1) out of the total of 82 families present in the PlnTFDB (26). Tests for TF-family enrichment were performed with the Fisher exact test. One-tailed Fisher exact *P*-values corresponding to the significance of overrepresentation of TFs belonging to the selected TF-family have been calculated based on counts in 2 × 2 contingency tables. Counts *n*_11_, *n*_12_, *n*_21_ and *n*_22_ in the contingency table refer to *n*_11_, number of correlated TF–TG pairs with TFs of a given TF-family; *n*_12_, number of correlated pairs with the TFs of other TF-families; *n_2_*_1_, number of uncorrelated pairs with the TFs of the particular TF-family; *n_2_*_2_, number of uncorrelated pairs with the TFs of other TF-families. Listed *P*-values correspond to multiple testing-corrected Fisher exact *P*-values using the Benjamini–Hochberg method, referred to as False Discovery Rate (FDR) throughout this article (27).

**Table 1.** List of TF-families enriched in correlated and uncorrelated TF–TG pairs for the set of active TFs

TF-family	Number of active TFs in TF-family	Number of TF–TG pairs created with active TFs from TF-family	Number of all TFs in TF-family	Fisher exact test, p_FDR_	Odds ratio
**TF-families enriched in correlated TF–TG pairs**					
**WRKY**	21	10 534	72	0.00E+00	3.74
**TCP**	5	531	24	4.83E-25	3.29
**MYB**	18	5627	147	5.54E–18	1.49
**HB**	5	1746	91	5.05E–04	1.31
**E2F-DP**	2	229	8	7.51E–03	1.70
**NAC**	5	1261	104	9.04E–03	1.26
**TF-families enriched in uncorrelated TF–TG pairs**					
**AP2-EREBP**	39	8590	146	1.38E–158	0.21
**C2C2-Dof**	5	3258	36	9.85E–43	0.31
**bZIP**	14	5510	70	1.45E–41	0.44
**BBR/BPC**	1	897	7	1.14E–29	0.06
**C2C2-GATA**	3	689	29	9.28E–29	0.00
**BES1**	4	1322	8	2.08E–19	0.29
**C3H**	1	702	68	1.56E–17	0.14
**LOB**	3	508	43	3.76E–13	0.14
**S1Fa-like**	1	261	3	3.20E–11	0.00
**CAMTA**	1	312	6	1.18E–10	0.06
**HSF**	6	840	23	5.02E–08	0.43
**bHLH**	2	217	136	4.40E–07	0.09
**C2H2**	3	917	99	8.95E–03	0.72

Significance was calculated based on the Fisher exact test for enrichment in the set of correlated pairs (*r* > 0.4) versus uncorrelated (–0.1 < *r* < +0.1) pairs with FDR correction for multiple testing. Odds ratios represent relative enrichment of counts in correlated versus uncorrelated pairs. Note that counts entering the enrichment statistic and computed *P*-values were based on TF–TG pairs, such that every TF entered the statistic based on all its TGs.

### GO enrichment analysis

Gene ontology (GO) annotation information, GO-slim process categories, was obtained from TAIR-10 database (21). Tests for GO-term enrichment were performed with the Fisher exact test as described above, but with GO-term-counts replacing TF-family-counts.

### Collected sets of features associated with expression regulation

#### TF and TFBS information

##### TFBS composition, entropy

The PCD provides information about TFBS motifs for every of the 386 profiled TFs in the form of a position weight matrix (PWM). The length of motifs, *N*, was taken as the number of positions in the PWMs (number of base pairs).

From the PWMs assigned to each TF, we calculated the percentage occurrences of each of the four different bases by summing the probabilities of each base-type across all positions in the PWM, and dividing it by the length of the PWM-motif. With the information about the frequency of each base type in a given TFBS motif, we calculated the ratio of A–T relative to G–C base pairs, i.e. double versus triple H-bonded base pairs, and likewise, A–G pairs relative to T–C base pairs, i.e. purines versus pyrimidines.

To test for specificity of TFBSs, we computed the sequence entropy (SE) of each TFBS motif. SE was defined as the average positional entropy (S) of a motif (Equation 1),

(1) $$\begin{eqnarray*} && SE = \frac{{\mathop \sum \nolimits_{i = 1}^N {S_i}}}{N}, \nonumber \\ && \quad \hbox{ with }{S_i} = - \mathop \sum \limits_{j \in \left( {A,C,G,T} \right)} {p_{i,j}}\ log\left( {{p_{i,j}}} \right), \end{eqnarray*}$$

where *p*_*i*,*j*_ is the relative frequency of a base *j* at position *i*, and *N* is the total length (number of basepairs) of a given TFBS. Similarly, the average sequence entropy associated with the five positions of lowest *S*-values was computed as well (core motif entropy), to account for potential problems of comparing SEs of TFBSs with different lengths. Here, five positions were chosen to capture a minimal TF–DNA core binding site.

##### Distribution of TFBS in promoters

For each TF–TG pair, the distance between the TFBS of the TF and the TSS of the TG was determined. In cases of multiple TFBS-occurrences, the distance of the TFBS closest to the TSS was taken. For every TF, we calculated the total number of TGs, and for TGs, we calculated the number of all TFs with TFBS in their promoter regions. For TGs, we calculated the average and standard deviation of the distance between TFBSs and the associated TSS for all TFs, which have TFBSs present in a distance interval of –1000 bp to +500 bp from the respective TSS. For TFs, we calculated the mean and standard deviation of the distance between associated TFBSs and TSSs across all TGs. Only TFBSs in a distance interval of –1000 bp to +500 bp from any TSS were used. Here, we considered a larger sequence interval around the TSS than the one used to identify candidate targets (–500 bp to –1 bp, equivalent to the upstream promoter) as a wider interval may be relevant for expression modulation as shown in *E. coli* (28). Distances between TFBS and TSSs were calculated by bedtools’ ‘closest’ function (22).

##### Hierarchical layers of processing

The set of regulatory TF–TG pairs was used to infer regulatory connections between TFs themselves, where TFs are assigned as TFs, but also as TGs of other TFs. From the network, we extracted information about the number of ‘in’ and ‘out’ edges for every TF, which stands for the number of regulators acting on the TF and the number of TFs regulated by this TF, respectively. The ratio of ‘in’ edges to all edges (‘in’ plus ‘out’) was calculated, referred to as ‘Number of TFs regulating the expression of TF over all connections of TF’. Similar to Duan *et al.* (29), this ratio allows assigning a relative position of a TF in the TF-regulatory network, considering initiation (top-level), processing, and effector (bottom-level) layers as conceptual layers of information processing. Ratios of or near zero indicate top-level TFs in the regulatory network hierarchy, while ratios near one indicate bottom-level TFs that have as targets genes other than TFs. Inference of network and all analysis were performed with ‘igraph’ package implemented in R (30).

### Post-transcriptional and post-translational modifications, including protein–protein interaction

#### miRNAs and associated target genes

Sequences of known miRNAs of *A. thaliana* were obtained from the miRBase v22.1 database (31). The miRNA-target search was done using as candidate targets (a) the cDNA sequence of all protein-coding genes, and (b) complete DNA sequences of genes, including introns, of *A. thaliana*. The inclusion of full-length gene sequences, i.e. including introns, was motivated by reports that miRNAs can also target introns and/or splice sites of nascent mRNA with a potential role in gene regulation through e.g. splicing regulation (32). Target prediction was performed using the TAPIR-Fast and TAPIR-Precise software (33). For both algorithms, the score cutoff was set to 4.0 and the free energy ratio to 0.7, where ratio refers to the fraction of the actual free energy gain upon miRNA–target interaction to the free energy gain of a perfectly matching miRNA–mRNA pair. The union of predictions generated by TAPIR-Fast and TAPIR-Precise was used as the set of miRNA and their target genes. The number of predicted miRNA target sites was determined for every gene, along with the maximal free energy ratio between gene and all miRNAs that can target this gene. From the predictions obtained from using full DNA sequences as potential targets, i.e. including introns sequence, we extracted only those interactions with miRNA, which targeted introns or exon-intron junction and calculated the number of those interactions for TFs and TGs as the number of target sites of miRNA targeting the introns in the pre-mRNA.

From the TAPIR-Precise algorithm, predictions of miRNA mimicry targets (34) were obtained. The number of predicted miRNA mimicry target sites was determined for every gene, using cDNA sequence, along with the maximal free energy ratio between gene and miRNAs, from the mimicry search.

#### mRNA stability

The stability of mRNAs was taken as reported in (35). The measurements of the mRNA decay were performed in *A. thaliana* cell cultures. The half-life, the time needed to degrade half of the molecules, of 13 012 mRNAs was calculated from nonlinear least-square regression fitted to experimental data. For every TF–TG pair, the product of half-lifes for the respective TF and TG was calculated. Values of the half-life associated with TGs, TFs and the product of those half-lives for every TF–TG pair were obtained from the supplementary results of (35).

#### Number of phosphorylation sites

The database PhosPhAt (36,37), version 4.0, was used as a data source for phosphorylation sites of TFs. Information about phosphorylation sites was obtained for 7959 proteins (including 112 TFs). For each TF, we collected the number of phosphorylation sites validated by experimental data. For TFs with different numbers of phosphorylation sites reported for different isoforms, the respective mean number of phosphorylation sites was taken.

#### Protein–protein interaction network

Information about protein–protein interactions (PPI) was obtained from APID (38), AtPIN_PPI (39), BIND (40), BioGrid (41), DIP (42), IntAct (43), Interoporc (44), iRefIndex (45), MINT (46), MolCon (http://www.ebi.ac.uk/Tools/webservices/psicquic/view/main.xhtml), STRING (47) databases. From all databases, we discarded connections supported by co-expression or text mining only. Taken as a superset of all data resources, we created a PPI network, in which nodes are proteins and edges represent interactions between two proteins present in the PPI databases. We created two different PPI networks; (i) built with the use of all proteins present in databases; (ii) built with the use of only TFs present in the PCD only. From the first PPI network, we calculated the shortest path between TFs of interest and the largest subunit of the Pol-II complex (TAIR ID: AT4G35800), as the PPI-distance between TF and the polymerase Pol-II subunit. The node degree of every TF was calculated for both PPI networks. Self-interactions, i.e. homo-multimers, were detected by a non-zero difference between the number of nodes with loops included and the number of nodes without loops, calculated for the first PPI network. If a TF from the PCD was not present in PPI data, we assigned an arbitrarily large value of ‘2000’ as the distance to Pol-II subunit and ‘0’ for all other parameters. If no valid pathway between TFs and Pol-II subunit was detected, reported as ‘Inf’, we assigned the value of ‘1000’ instead. Inferences of PPI networks and all analyses were performed with the ‘igraph’ package implemented in R (30).

### Genomic and genome annotation derived information

#### Genomic data about genes

Genome annotation information and all information pertaining to positions of genomic elements used below were obtained from the available TAIR-10 (21) annotation. From the GFF file, we extracted information about the number of isoforms, lengths of mRNAs, protein, 3′UTR and 5′UTR for all the present isoforms, and distance to the closest upstream gene. For every gene, we calculated the maximum, minimum, and mean length of those selected features for all present isoforms and difference of the protein length. For 5′UTRs, information about the presence of introns was extracted. We called 5′UTR as intron-containing, if there was an overlap between the 5′UTR and an intronic region. The number of isoforms for each gene was determined, as well as the difference between the shortest and the longest protein (splice variants) encoded by each gene. A set of 7679 genes, including 59 TFs, with missing information of 5′UTR length (5′UTR length was equal to 0) was removed from the analysis.

All computations for detecting overlaps of genomic regions were performed using bedtools’ ‘intersect’ function (22).

#### Evolutionary age of genes

The evolutionary age of genes was taken from a previous study that assigned *A. thaliana* genes to 13 different phylostrata representing different evolutionary ages. Based on BLAST-based protein sequence homology searches against species of different evolutionary age, *A. thaliana* genes were assigned to the respective oldest age bin with a significant BLAST-hit (48). Accordingly, *A. thaliana* genes were binned into 13 phylostrata, PS1 to PS13. Oldest genes, with homologous sequences in prokaryotes, were assigned to PS1, and youngest genes, those without any homologs in any species other than *A. thaliana*, were assigned to PS13. All other categories constitute genes of gradually evolutionarily younger genes from PS1 to PS13. Those categories were assigned to all TGs and TFs in our dataset. For each TF–TG gene pair, the difference of evolutionary age was calculated as the difference between evolutionary age category of the TG and evolutionary age category of the regulating TF (age_TG_–age_TF_).

#### DNA binding domains

From the Uniprot database (49), we extracted information about the first and, if present, second DNA-binding domain (DBD) for every TF, where ‘first’ and ‘second’ relates to ordering relative to the N-terminus of the TF-protein. We searched the Uniprot database with AGI names for each TF, and from the results, we extracted information about the length of the respective protein, number of DBDs, with value 1 assigned to TFs with only one DBD, and 2 assigned to TFs with two or more DBD, starting position of a DBD for the first and the second DBD, if present. Then, we calculated the relative distance of the starting position of each DBD to the N-terminus of the protein, measured by the ratio of the starting position of DBD relative to the N-terminus and the length of the protein.

#### Promoter composition

Promoter composition of every gene was calculated as a percentage composition of each nucleotide, A, C, G and T, and also counts of each of the 16 possible dinucleotides. The number of dinucleotides was calculated with the use of the sliding window of length 2 with a stepsize of one nucleotide in the promoter DNA sequence. The promoter region was obtained from TAIR-10 (21) as a region of –500 bp to –1 bp relative to the TSS and promoter sequence was obtained from FASTA file for TAIR-10 (21) with use of bedtools’ ‘getfasta’ function (22).

#### TATA-box in gene promoters

Genes were considered TATA-box containing, if they contained a TATA-box motif (consensus sequence ‘TATAWA’ (50) in the 60bp upstream of the TSS. The TATA-box motif was searched in forward orientation only, as for the TATA-box motif, orientation-dependence has been reported with only the forward motif being active (51). In total, 4739 genes, 4185 present in the PCD, were considered TATA-box-containing and were profiled with regard to expression (on the ATH1-Affymetrix microarray, requiring uniqueness of ATH1-probe/ AGI-identifier mapping).

### Epigenetics

#### DNA methylation

Information about differentially methylated regions of DNA was obtained from two sources: the 1001 Genome Project (52) and from (53). From the 1001 Genome Project, methylomes were collected for 1107 different *A. thaliana* accessions. From (53), information on 152 methylomes of different *A. thaliana* accessions from the Northern hemisphere was obtained. In each dataset, the localization of differentially methylated regions was reported for the CpG and the CHH sequence context, where H is any nucleotide except G, and in the 1001 Genome Project dataset, also for the CHG context. The number of differentially methylated regions present in the selected genomic regions was calculated in every promoter of the TG, defined as –500 bp to –1 bp interval relative to the TSS; regions around the TSS, –100 bp to 100 bp from TSS, and gene body region.

#### Open chromatin marks

The localization of open chromatin marks, identified by DNase-seq as DNase-I Hypersensitive Sites (DHS), was obtained from (54), reported for plants during photomorphogenesis and heat stress, and from (55), DHS regions obtained from wild type plants from flowers and leaves. 734 and 1980 different DHS were reported after light stimuli and after heat stress, respectively. 38 290 and 41 193 different DHS were reported in leaves and flowers, respectively. From those four datasets covering different conditions and different tissues, the localization of stable and dynamic DHS were obtained. Stable DHS were defined as DHS present in all samples, i.e. intersection of all four datasets. Dynamic DHS were defined as DHS present in at least one sample, but not all samples, i.e. union of all four datasets without stable DHS minus the intersection set (exclusive OR). Note that with regard to stable DHS, we operated under the assumption that if DHS were found consistently across four very different conditions, they can also be assumed open under different conditions, including those covered by the used expression data. We found 24 845 stable DHS and 69 856 dynamic DHS. For each TG, we calculated the number of stable and dynamic DHS present in their promoter region, defined as –500 bp to –1 bp sequence interval relative to the TSS, and in the gene body region.

The information about stable and dynamic DHS was merged with the information about the localization of TFBSs in promoter regions. For each regulatory TF–TG pair, binary information about the presence of TFBS in the stable or dynamic DHS was generated, where value ‘1’ was assigned to all pairs with TFBS in the stable or dynamic DHS and value ‘0’ assigned to all pairs without TFBS in the stable or dynamic DHS.

#### Number of epigenetic mark types

The percentage of epigenetic mark types for each gene was calculated as the ratio of the number of epigenetic marks: differentially methylated DNA regions, and open chromatin regions, present for this gene over the total number of all potential epigenetic mark types.

### Information about adjacent genes

Adjacent protein-coding genes were selected as genes with a distance equal or smaller to thresholds of 0.5, 1, 2, 5 kb, from the positional boundaries of a gene of interest. From the subset of genes assigned as adjacent, we calculated their mean expression based on the 5296 microarray hybridizations, mean number of TFBS in their promoters, and the number of shared TFBS with the gene of interest. List of adjacent genes was obtained by bedtools’ ‘closest’ function (22).

### Imputation and scaling

Imputation of missing data was performed with the use of the ‘mice’ package (56). Imputation was done on the R-dataframe with information about TF and separately on the dataframe with information about all TGs.

To collapse highly correlated features in our model, we standardize all features to a distribution with mean = 0 and SD = 1 and clustered them based on the Pearson correlation coefficient. Correlated features (Pearson correlation coefficient above 0.85) were merged (average) and treated as one feature. From the set of 135 features, 112 clusters of features were created treated as the predictor variables.

### Univariate testing of feature importance, effect size

For every considered molecular feature, the significance of differences between the set of correlated and uncorrelated TF–TG pairs was assessed by applying the non-parametric Wilcoxon–Mann–Whitney test as implemented in R, and the magnitude of difference (effect size) quantified as Cohen's effect size, *D*, as implemented in the R package ‘effectsize’ R (57). In essence, Cohen's *D* captures the difference of two means relative to the average standard deviation of the observations.

### Random Forest classification

#### Random Forest (RF) model

The input dataset used for the RF model was created for all 104 369 regulatory pairs obtained from PCD, created between TFs assigned as ‘active’ and TGs with annotated 5′UTR. Regulatory pairs were divided into two sets: correlated pairs, with a Pearson correlation coefficient >0.4, and uncorrelated, with Pearson correlation coefficient greater than −0.1 and smaller than 0.1. Removing all other pairs resulted in a decrease of the number of pairs from 104 369 to 48 090 . Using as categorical outcome variables the labels ‘correlated’ or ‘uncorrelated’, we applied the Random Forest classifier, with 3000 trees and five variables selected for each tree, with the number five selected to correspond to 0.5 × sqrt(number of feature clusters), on our dataframe with a total of 112 clustered predictor variables, and with minimal size of the terminal nodes of 150 observations. By choosing high numbers of observations in terminal nodes, i.e. not further splitting the data to purity, and low numbers of variables to enter each tree, our settings were intended to reduce overfitting. Random Forest classification was used as implemented in the ‘randomForest’ R package (58).

#### Cross-validation

From the set of TFs and all potential TGs, we selected two subsets, a training and a test set. In each subset, a non-overlapping set of TFs and TGs were present, i.e. sets were rendered non-redundant not only with regard to pairs, but pair-members as well. Not implementing this strict check for non-redundancy and basing it on the pair-identity alone, bears the risk of memorizing the properties of a pair member, leading to overly optimistic results as it is recognized again in the test set. We randomly selected 80% of TFs and TGs to be present in the training set and the remaining 20% present in the test set. With this split, the RF machine learning model was trained only on pairs formed by TFs and TGs contained in the training subset. TF–TG pairs formed between TFs and TGs from the respective other set (training/ testing) were discarded. It is important to note that we report performances not as OOB (out-of-bag) error as done typically for RF, but used cross-validation that guaranteed absence of overlap between any of the components (TFs, TGs) between the training and test set. Created datasets have high class imbalances, only eight percent of all pairs were assigned to the class ‘correlated’. To eliminate imbalanced-classes problem, we undersampled the majority group, i.e. uncorrelated pairs, to have an equal number of samples for both classes. The procedure of random sampling, fitting of RF, and performance evaluation was performed 20 times. For better execution performance, each RF was trained with the use of the ‘foreach’ function, with the ‘doParallel’ backend, on six cores with the forest of 500 trees to train for each core, which were further combined into one random forest model with 3000 trees in total (59,60).

#### Classification performance assessment and variable importance estimation

The performance of the RF-classification was measured after each of the 20 Random Forest training runs. The performance was measured by means of the area under the curve (AUC) for the receiver operating characteristics (ROC) and the precision–recall (PR) curve. Both measurements were performed on the respective test subset results. Predictions for pairs from the test set were compared with the true labels for those pairs. AUC scores were calculated with R package ‘PRROC’ (61).

Variable importance was measured as the mean decrease of accuracy (MDA) for each variable after every iteration of Random Forest training. Mean values of MDA from all iterations were used as a final score for feature importance. MDA score was calculated using the ‘randomForest’ R package (58).

#### Interaction of features

Feature interactions were assessed by using RandomForestExplainer (62). The basic rationale implemented in this method posits that interactions between variables (features) become evident as frequent occurrences of close distances between them in the hierarchy of splits of the classification trees grown as part of a RF model. All variables of the model are each considered as conditioning variables. Then, for every variable—taken as the conditioning variable—the frequency and distances to all other variables (including the considered conditioning variable) are recorded for all RF tree models and the most frequently occurring pairs of variables and their average distances reported. Here, frequency is taken as a number of trees in which a particular variable pair was contained as part of the same subtree and with the conditioning variable closer to the root of the tree than the respective other variable. The observed distances are compared to the observed unconditional distances of all variables to the root (first split) across all trees. If interactions are relevant, the unconditional distances of all variables should be larger than the distances to conditioning variables, with which it is interacting. Interactions also become evident as frequent occurrences of variable combinations. Thus, both, the pair frequency and the associated distances are informative with regard to interaction effects. The reported interaction effects have been obtained as averages over all 20 performed cross-validation runs.

#### Data availability

All TF–TG pairs, along with all feature annotations for all genes present in them, is available as Supplementary information (Supplementary Table S3).

## RESULTS

### Influence of transcription factors (TFs) on the expression of their target genes (TGs)

The PCD provides information about transcription factor (TF) binding events to genomic DNA and derived binding sites (TFBSs) of 386 TFs, obtained from DNA affinity purification sequencing (DAP-seq) assays applied in the plant *A. thaliana*. Requiring TFBSs to be located in the upstream promoter region of a potential target gene (TG), with promoters assumed as the interval of –500 bp to –1 bp of a gene's transcription start site (TSS), we identified a set of 280 655 regulatory pairs of TFs and their candidate TGs. Inspecting the associated correlations of gene expression of all such TF–TG pairs across a large dataset of microarray-based gene expression profiling experiments, only a small, albeit significant, shift toward larger positive values was evident, when comparing those assumed true TF–TG pairs to pairs of TFs and randomly chosen non-TGs (Figure 1A). The goal of this study is to determine whether this weak evidence of transcriptional coupling applies uniformly to all TF–TG pairs or whether particular molecular properties render some TF–TG pairs transcriptionally coupled, while others are under more complex regulatory control mechanisms, perturbing the direct correlation between expression of TFs and their TGs.

Some of the DAP-seq profiled TFs may exert only a small effect on their TGs or only under very few conditions probed as part of the available gene expression datasets. Thus, such TFs would not lead to a discernable expression-mediated regulatory effect in our expression dataset. Alternatively, the TF may very well have an effect, which, however, cannot be related to its expression level, but is connected to other factors such as protein level, post-translational modification, other interaction partners etc. While the former explanation would be a technical limitation, the extent of the latter is one central question of this study. Hence, we first sought to identify those TFs with a noticeable and coherent effect on altered gene expression of its TGs and independently of the mechanism of regulation of TF-activity. As evidence of TF-activity, we probed for pairwise gene expression correlation among candidate TGs, predicted from the DAP-seq dataset, i.e. we estimated the activity of a TF, based on its effect on its TGs as done similarly before (63,64). The distribution of correlation coefficients computed among TGs proved significantly shifted to larger positive values compared to correlation levels of randomly chosen genes (Figure 1B). Thus, TF-activity was, in fact, evident in the data, and furthermore, we can distinguish those with an effect (called ‘active’ TFs) from those that do not show any discernible evidence of activity in our expression dataset. Consequently, from the set of all TFs, we selected as active all those, for which the expression of correlation among all TGs was greater than the 97.5th percentile level of mean correlation values of non-TGs (mean Pearson correlation between TGs expression, *r* > 0.013; Figure 1B). We obtained a set of 157 TFs considered active as evidenced by an associated correlated expression of their TGs.

For the 157 TFs considered active, we created the associated set of regulatory TF–TG pairs (104 369 pairs in total). The correlation levels between active TFs and their TGs was significantly higher relative to considering all regulatory pairs based on all 386 TFs (*P*-value = 2E–39, Wilcoxon–Mann–Whitney test) (Figure 1A and C). A more pronounced difference was observed between TF–TG pairs and TF–non-TG pairs, when considering active TFs only (*P*-value = 5.8E–205, Wilcoxon–Mann–Whitney test; Cohen's *D* effect size = 0.16) compared to the respective comparison for all TFs (*P*-value = 2.9E–143, Wilcoxon–Mann–Whitney test; effect size = 0.07). Nonetheless, the large overlap between true and false regulatory pairs still precludes using co-expression alone as evidence of a regulatory relationship.

In the following, we aim to identify the determinants of direct transcriptional coupling as opposed to the more complex regulation of gene expression regulation for this set of 157 active TFs and their TGs.

### Selected TF-families are enriched in the set of TFs with high correlation with their TGs

It appears possible that direct transcriptional coupling between TFs and their TGs, resulting in correlated gene expression, is evident for particular TF-families only, operating with particular modes of action. Based on their domain architecture, TFs present in *A. thaliana* can be classified into 82 TF-families (26) with members of 24 families found present in the set of selected active 157 TFs (Supplementary Table S1). Indeed, six TF-families (WRKY, TCP, MYB, HB, E2F-DP, NAC) were found significantly enriched (*p*_FDR_ < 0.01, Fisher exact test) in the set of TF–TG pairs with pronounced expression correlation (Pearson correlation coefficient, *r*, with *r* > 0.4; 4340 pairs, 4.2% of the total set) relative to a set of uncorrelated pairs (–0.1$ <$*r*$ <$0.1; 43 750 pairs, 41.9% of the total set, Figure 1C) (Table 1). In the set of TF–TG pairs, which were considered uncorrelated, an enrichment of 13 TF-families was observed (AP2-EREBP, C2C2-Dof, bZIP, BBR/BPC, C2C2-GATA, BES1, C3H, LOB, S1Fa-like, CAMTA, HSF, bHLH, C2H2) (Table 1). As this overrepresentation of particular TF-families in the correlated as well as uncorrelated pair set must have underlying molecular causes, those results imply that indeed, there are characteristic molecular determinants of high or low gene expression correlation between TFs and their TGs.

### Correlated pairs are enriched for selected molecular processes

We performed a GO (gene ontology)-term enrichment analysis on the correlated pair set to determine, which molecular processes rely on direct transcriptional coupling between TFs and their TGs. TFs and TGs present in correlated pairs show high enrichment in categories associated with response mechanisms to external and dynamic environmental perturbations (‘response to external stimulus’, ‘response to stress’, ‘response to abiotic stimuli’, and ‘response to biotic stimuli’) (Table 2). To a lesser degree, albeit significant, TFs and TGs forming correlated TF–TG pairs were also found enriched for GO-categories associated with regulation of ‘embryo development’, ‘cell cycle’ and ‘cell death’, processes that are more developmental and ‘house-keeping’ in nature. Thus, direct transcriptional coupling appears to be more relevant for processes associated with a response to the external stimuli, both biotic and abiotic, rather than developmental programs.

**Table 2.** List of Gene Ontology (GO) process categories enriched in correlated and uncorrelated TF–TG pairs for active TFs and their TGs

Transcription factors			Target genes		
GO category	Fisher exact test, *p*_FDR_	Odds ratio	GO category	Fisher exact test, p_FDR_	Odds ratio
**Enriched in correlated TF–TG pairs**					
**response to external stimulus**	**7.17E−92**	**2.04**	**response to biotic stimulus**	**2.31E−85**	**2.67**
**response to stress**	**3.15E−74**	**1.52**	**response to external stimulus**	**1.47E−69**	**2.25**
**response to biotic stimulus**	**2.39E−69**	**2.03**	**response to stress**	**5.90E−64**	**1.72**
**catabolic process**	**4.68E−50**	**4.27**	**response to chemical**	**2.03E−36**	**1.57**
**response to abiotic stimulus**	**1.62E−35**	**1.40**	**response to abiotic stimulus**	**3.24E−23**	**1.55**
**cell cycle**	**9.73E−23**	**3.17**	**secondary metabolic process**	**2.32E−18**	**2.10**
**cellular homeostasis**	**9.00E−12**	**2.67**	**cell death**	**7.29E−13**	**2.42**
**cell communication**	**2.76E−10**	**1.46**	**photosynthesis**	**1.48E−07**	**1.90**
**embryo development**	**1.17E−06**	**1.70**	**response to endogenous stimulus**	**3.07E−07**	**1.29**
**regulation of molecular function**	**5.28E−04**	**1.41**	**cellular protein modification process**	**6.27E−07**	**1.24**
**response to chemical**	**6.65E−04**	**1.07**	**signal transduction**	**7.48E−04**	**1.19**
transport	3.45E−02	1.32	cell communication	1.39E−02	1.23
lipid metabolic process	4.68E−02	1.29	generation of precursor metabolites and energy	1.88E−02	1.28
secondary metabolic process	9.51E−02	1.10	abscission	6.67E−02	1.81
cell differentiation	2.41E−01	1.04	fruit ripening	1.05E−01	3.81
other biological processes	3.03E−01	1.05	catabolic process	2.05E−01	1.06
growth	3.03E−01	1.05	RNA binding	2.38E−01	8.89
DNA binding	3.05E−01	1.07	lipid binding	2.38E−01	8.89
nucleic acid binding	3.05E−01	1.07	regulation of molecular function	5.03E−01	1.02
response to endogenous stimulus	3.64E−01	1.01	DNA-binding transcription factor activity	5.76E−01	1.48
			other binding	5.76E−01	1.48
			catalytic activity	6.79E−01	0.99
**Enriched in uncorrelated TF−TG pairs**					
**response to light stimulus**	**9.90E−46**	**0.41**	**cellular component organization**	**1.18E−35**	**0.53**
**signal transduction**	**1.55E−36**	**0.65**	**reproduction**	**1.32E−19**	**0.55**
**unknown biological processes**	**9.33E−16**	**0.00**	**unknown biological processes**	**1.53E−19**	**0.68**
**nucleobase-containing compound metabolic process**	**3.66E−13**	**0.88**	**nucleobase-containing compound metabolic process**	**1.71E−15**	**0.70**
**biosynthetic process**	**3.66E−13**	**0.88**	**DNA metabolic process**	**6.44E−13**	**0.37**
**other metabolic processes**	**3.66E−13**	**0.88**	**post-embryonic development**	**2.73E−12**	**0.62**
**other cellular processes**	**3.66E−13**	**0.88**	**growth**	**2.43E−11**	**0.48**
**abscission**	**2.63E−11**	**0.23**	**anatomical structure development**	**3.54E−10**	**0.73**
**flower development**	**7.56E−10**	**0.66**	**transport**	**2.08E−09**	**0.73**
**protein binding**	**1.62E−09**	**0.20**	**cell differentiation**	**5.89E−09**	**0.56**
**tropism**	**6.87E−08**	**0.28**	**cell growth**	**2.43E−08**	**0.49**
**post-embryonic development**	**6.07E−05**	**0.84**	**flower development**	**2.43E−08**	**0.53**
**cell death**	**8.65E−04**	**0.64**	**multicellular organism development**	**2.43E−08**	**0.76**
**fruit ripening**	**8.65E−04**	**0.38**	**protein metabolic process**	**2.51E−08**	**0.70**
cellular component organization	2.08E−03	0.73	**regulation of gene expression, epigenetic**	**8.14E−08**	**0.26**
cell growth	4.68E−02	0.82	**cell cycle**	**3.72E−07**	**0.56**
reproduction	1.35E−01	0.93	**circadian rhythm**	**1.20E−06**	**0.39**
multicellular organism development	1.51E−01	0.96	**biosynthetic process**	**2.50E−06**	**0.84**
circadian rhythm	2.03E−01	0.89	**embryo development**	**3.76E−06**	**0.62**
photosynthesis	2.78E−01	0.89	**carbohydrate metabolic process**	**1.45E−04**	**0.76**
anatomical structure development	2.78E−01	0.97	**lipid metabolic process**	**4.47E−04**	**0.76**
carbohydrate metabolic process	3.64E−01	0.93	cellular homeostasis	2.34E−03	0.66
			tropism	3.67E−02	0.53
			other cellular processes	4.07E−02	0.95
			cell−cell signaling	1.14E−01	0.34
			other metabolic processes	2.12E−01	0.98
			response to light stimulus	2.13E−01	0.92
			other biological processes	2.38E−01	0.93
			translation	3.54E−01	0.94
			pollination	4.40E−01	0.94
			protein binding	4.84E−01	0.88
			nucleic acid binding	6.79E−01	0.00
			DNA binding	6.79E−01	0.00
			transferase activity	8.99E−01	0.00
			transporter activity	8.99E−01	0.00

Significance was calculated based on Fisher exact tests for enrichment in the set of correlated pairs (*r* > 0.4) versus uncorrelated (−0.1$ <$*r*$ <$+0.1) TF–TG pairs with FDR correction for multiple testing. Highlighted in the bold-face font are terms with *p*_FDR_<0.001.

### Biological and molecular properties of TFs and their TGs considered as determinants of transcriptional coupling

Prompted by the found significant enrichment of particular TF-families and GO-categories in the set of highly correlated regulatory pairs, we looked for the specific gene-related molecular features of TFs and of TGs, which could be associated with high or low expression correlation, respectively. In essence, we wished to determine, which molecular features render regulatory pairs directly coupled via gene expression, and which ones are more likely characteristic of more complex regulatory processes. We aimed to select features related to the regulation of the activity of TFs as well as properties of the promoters of TGs, along with more general genomic properties. Based on publicly available datasets, we collected a total of 135 parameters that characterize TFs, their TGs and regulatory pairs (Supplementary Table S2). Selected attributes can be grouped into the following four categories: information about TFs and its TFBS, post-transcriptional and post-translational regulation (including protein–protein interactions (PPI)), genomic information and genome-derived annotation information, and epigenetics.

The found significant enrichment of particular TF-families in the set of correlated TF−TG pairs (Table 1) suggests a relevance of features directly associated with TFs and their mode of action. From the PCD, we derived information about the genomic localization of TF-binding events along with the number of potential TGs and the composition of TFBS-motifs, i.e. nucleotide composition and sequence entropy of the TFBS motif measured as the compositional entropy, SE (Equation 1). The localization of the TFBS in relation to TSS was calculated for each regulatory pair and also taken as averaged across all TGs as well. With the known potential regulatory connections between TFs and their TGs, we recreated the regulatory network from which we extracted network-related characteristics such as the number of regulatory connections and number of TFs, which are regulating a particular gene.

Post-transcriptional silencing and degradation of mRNAs through interaction with miRNAs, post-translational modification and interactions with other proteins can strongly influence the activity of the TFs (14,63,65–68). With the known sequences of miRNAs present in the *A. thaliana* genome, we predicted binding sites and the strength of the interaction between miRNAs and mRNAs, and also with pre-mRNA, including mimicry interactions (34). As for factors that influence the activity of TFs post-translationally, we selected information on the number of phosphorylation sites and information about protein−protein interactions (PPI). Based on the reconstructed PPI network, we probed for significance of the number of PPI and calculated the shortest paths between TFs and the largest Pol-II subunit, i.e. the theoretical number of interacting proteins needed to establish this interaction, allowing us to test whether short PPI-paths allow more direct transcriptional coupling.

The gathered genomic features and genome-derived annotation parameters such as the length of mRNAs and respectively encoded proteins, length of 5′UTR and 3′UTR, presence of introns in the 5′UTR, the number of splice variants described for genes, length of upstream intergenic regions, presence of a TATA-box in the promoter region, and the number of introns, have been described before to influence the expression response (18), and were included here as well. Additionally, we collected information about promoter regions such as nucleotide and dinucleotide composition. As genes can be arranged into transcriptional clusters on chromosomes not only in prokaryotes, but also in eukaryotes (69), the regulatory landscape of genes adjacent to TG of interest may contain information on its expression regulation and was included by various parameters (distance to the next upstream gene and others). For TFs, information about the domain architecture was compiled, i.e. the number of DNA binding domains and their localization in relation to the N-terminus. Based on the sequence similarity to proteins in other species, *A. thaliana* genes were grouped into 13 classes reflecting their evolutionary age ranging from the oldest genes having homologs in unicellular organisms, to the youngest, that are unique to *A. thaliana* (48), allowing us to test the hypothesis that direct transcriptional coupling may be associated with newly evolved genes, while more complex regulation has evolved for older genes.

Epigenetic markers, such as state of openness of the chromatin and methylation of DNA, can strongly influence the expression of nearby genes (54,70,71). For genes considered TGs, information about epigenetic markers, such as open chromatin sites, defined as DNase-I hypersensitive site (DHS), and differentially methylated DNA regions present in their promoter and gene body regions was collected. For each regulatory pair, we tested whether the TFBS of the TF was located in a region, which is regulated by changes of chromatin openness, i.e. was it located in the stable or dynamic DHS or neither.

A detailed list of all 135 features is contained in Supplementary Table S2 and described in Materials and Methods.

### Identification of feature importance based on univariate testing and Random Forest machine learning classification

**Figure 2.**
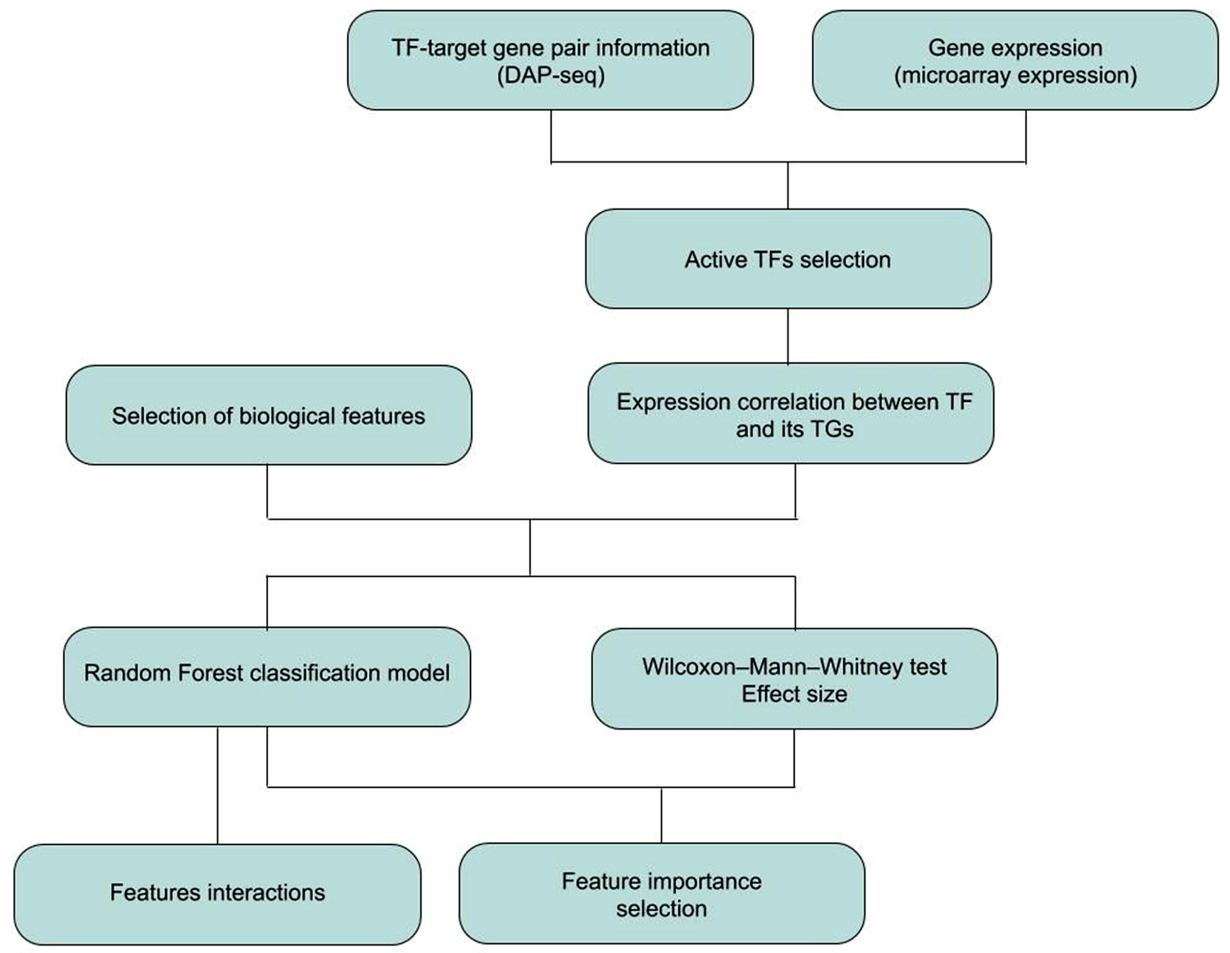
Flowchart describing the approach to feature selection associated with correlated TF−TG pairs. For each TF, information about binding events to genomic DNA was obtained from the Plant Cistrome Database (PCD) and candidate target genes (TGs) determined. Based on the expression information from a set of microarray expression hybridizations, the regulatory activity of each TF was calculated, yielding a set of 157 TFs considered active. For this set, the correlation of expression was calculated for each TF−TG regulatory pair. A set of 135 molecular features, consisting of information about TFs and their transcription factor binding sites (TFBSs), regulation of activity via post-transcriptional and post-translational modifications, including protein−protein interactions, genomic features, and modes of epigenetic regulation were tested whether they are associated with an increased correlation of regulatory pairs. To avoid the problem of correlated features, all biological features were clustered into 112 feature clusters. For each cluster, the difference between highly correlated and uncorrelated pairs was assessed based on the Wilcoxon−Mann−Whitney test and Cohen's *D* effect size. To evaluate more complex feature characteristics, analysis of feature importance was performed using the Random Forest classification algorithm. From this model, information about the importance of each feature was selected, along with information about features interactions.

To establish, which features were strongly associated with a direct transcriptional coupling between TFs and their TGs, all regulatory pairs associated with the set of the detected 157 active TFs were divided into two sets: correlated and uncorrelated pairs as described above (testing TF-family enrichment). To examine the importance of each feature, we performed univariate statistical testing of each feature between pairs in the correlated and the uncorrelated sets, and secondly, feature importance extraction from the classification machine learning algorithm, Random Forest. A schematic overview of the used data resources and performed analyses leading to these results are presented in Figure 2.

As some of features show high correlation or redundancy, e.g. length of the protein and length of the mRNA, we aggregated correlated features to avoid the problem of highly correlated features, which could influence our analyses, especially the feature importance extraction using the Random Forest machine learning algorithm. Aggregation of features resulted in a decrease of the number of features tested from 135 to 112.

Out of the 112 effective features, 28 features have absolute effect size >0.15 and 26 were also significantly different (adjusted *P*-value < 0.001) between correlated and uncorrelated pairs (Figure 4, Supplementary Table S4).

To identify more complex, possibly non-linear relationships between features, which can be missed by the univariate testing approach, we applied the Random Forest (RF) classification machine learning algorithm. Our model uses the selected 112 features to classify regulatory pairs of TFs and their TGs into either correlated or uncorrelated class. First, we wanted to estimate to what extent gene and genome-related features determine whether TFs and their TGs exhibit co-transcriptional coupling as evidenced by the correlated expression, and secondly, if yes, which features prove to be informative (feature selection).

**Figure 3.**
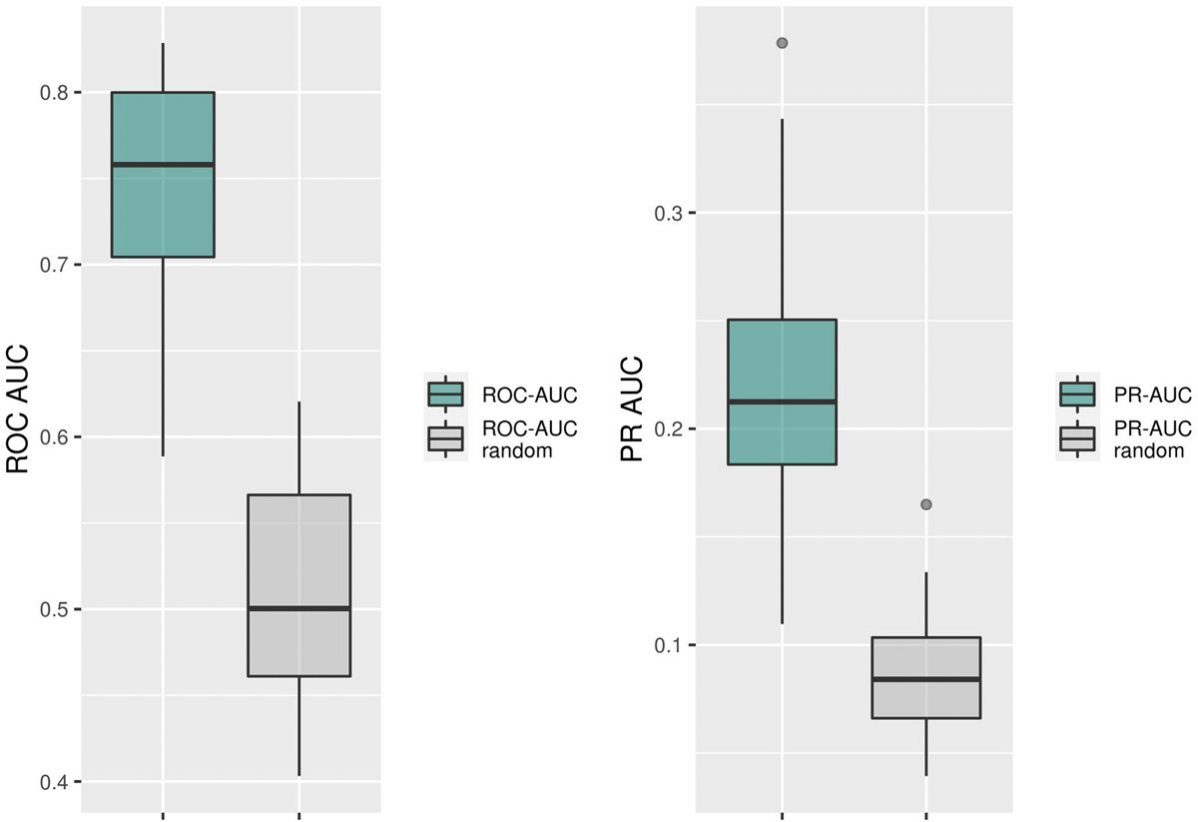
Performance of the Random Forest classification of TF−TG pairs into the correlated or uncorrelated class based on the collected set of features, measured by AUC of the ROC and PR curve. Green boxes represent the distribution of AUC values for prediction of the Random Forest model and grey boxes represent the distribution of AUC values of permuted class-labels. Values obtained from 20 times cross-validation runs. Median value for ROC−AUC = 0.76 and ROC-AUC (random) = 0.50. Median value for PR-AUC = 0.21 and PR–AUC (random) = 0.08. Values of ROC-AUC and PR-AUC obtained in our model were significantly higher than values obtained in permuted class-labels dataset, *P*-value = 1.0E−10 and 2.8E−10, Wilcoxon–Mann–Whitney test, respectively.

**Figure 4.**
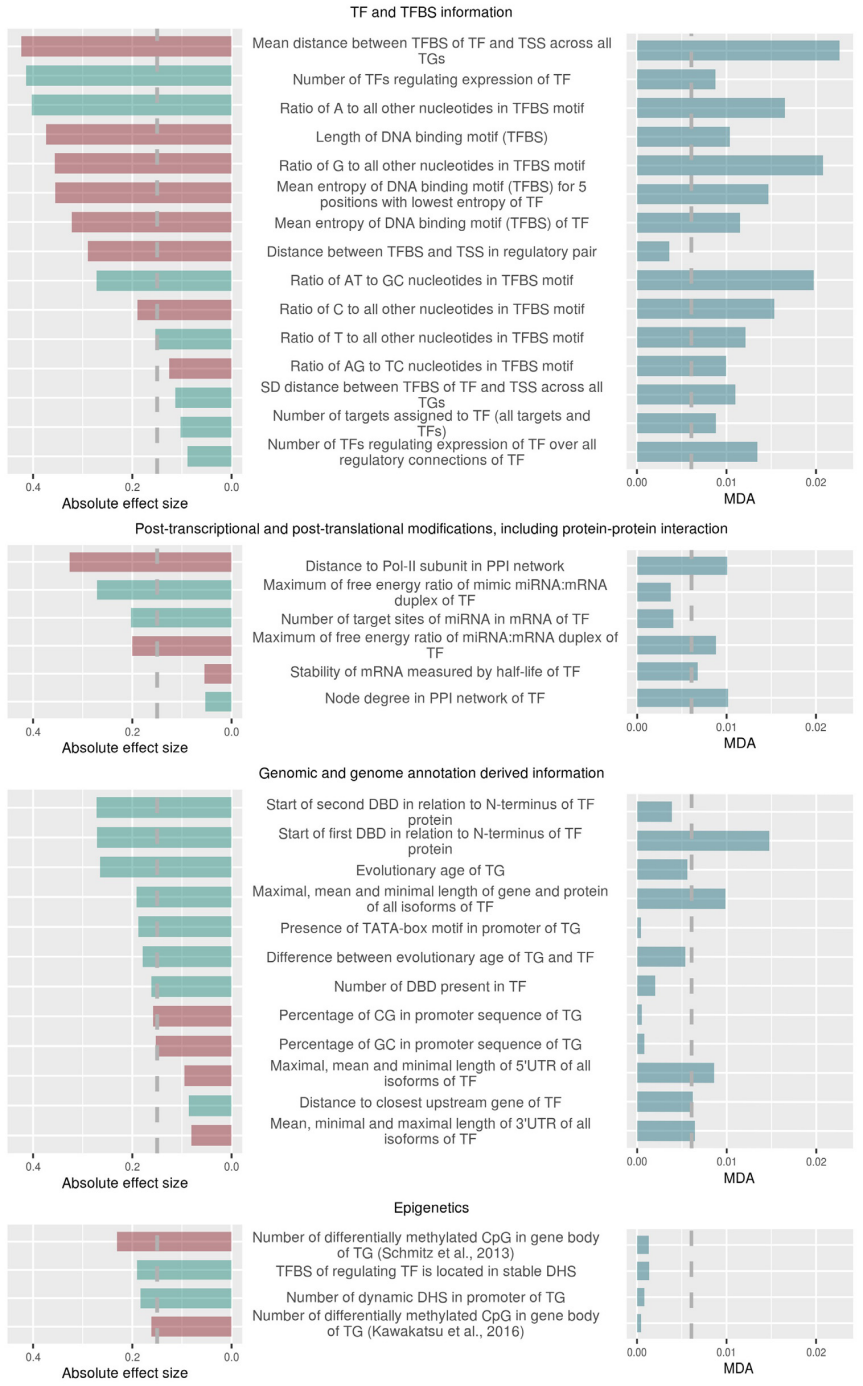
Feature effect sizes and importance in the Random Forest classifier distinguishing between correlated and uncorrelated pairs. The left panel presents the absolute effect size (Cohen's *D*) between correlated and uncorrelated regulatory pairs. Features with green/red bars show higher/lower values in correlated compared to uncorrelated pairs. Right panel presents MDA that results from a permutation of the selected feature in the Random Forest after training, but before prediction. Vertical dashed lines present arbitrarily chosen thresholds for absolute effect size (0.15) and for MDA (0.006), used to identify the most important features. Shown are the 37 most informative features out of the total of 112 tested features, taken as the union of most significant effect size variables and MDA. Significance at the level of *p*_FDR_<0.01 was observed for 33 features, features without statistical significance: ‘Number of target sites of miRNA in mRNA of TF’, ‘Stability of mRNA measured by half-life of TF’, ‘Maximum of free energy ratio of miRNA:mRNA duplex of TF’, ‘Mean, minimal and maximal length of 3′UTR of all isoforms of TF’.

Applying a rigorous testing scheme (see Materials and Methods), the classification of TF−TG pairs to belong to either the correlated or uncorrelated class proved possible. The median area under the ROC curve (ROC−AUC) was obtained as 0.76 (SD = 0.061) and the median area under the precision–recall curve (PR-AUC) was 0.21 (SD = 0.084) (Figure 3). Compared to randomly permuted data, those classification performance metrics indicate that our model performs significantly better than randomly guessing (Figure 3). Thus, with the set of selected features, we can predict with high confidence, whether a TF and its TG will exhibit expression correlation. The considered features used for making those predictions were indeed informative with regard to deciding whether a TF−TG pair will be coupled co-transcriptionally. The importance of features in the RF model was measured with the MDA after permutation of each parameter. Out of the total of 112 features, we selected the 23 most informative features, 20% of all features, with the highest MDA score (features with a MDA score > 0.006) (Figure 4).

### Features associated with expression coupling of regulatory pairs

The univariate testing identified 28 features that exhibit significant statistical differences and large absolute effect sizes, 14 of which were also found by the RF method as judged by MDA, constituting a significant overlap (*P*-value = 3.6E−5, Fisher exact test; odds ratio = 8.1). Nine features were selected exclusively by the RF algorithm. The respective performance metrics (absolute effect size and MDA) of the 37 features selected as important by at least one of the two approaches were found correlated (Pearson correlation coefficient, *r* = 0.41, *P*-value = 0.01). Thus, while both methods commonly identified identical features, each revealed specific features as well (Figure 4). The comprehensive overview of the results of all considered features are available as Supplementary Figures S3−S6, and Table S4.

**Figure 5.**
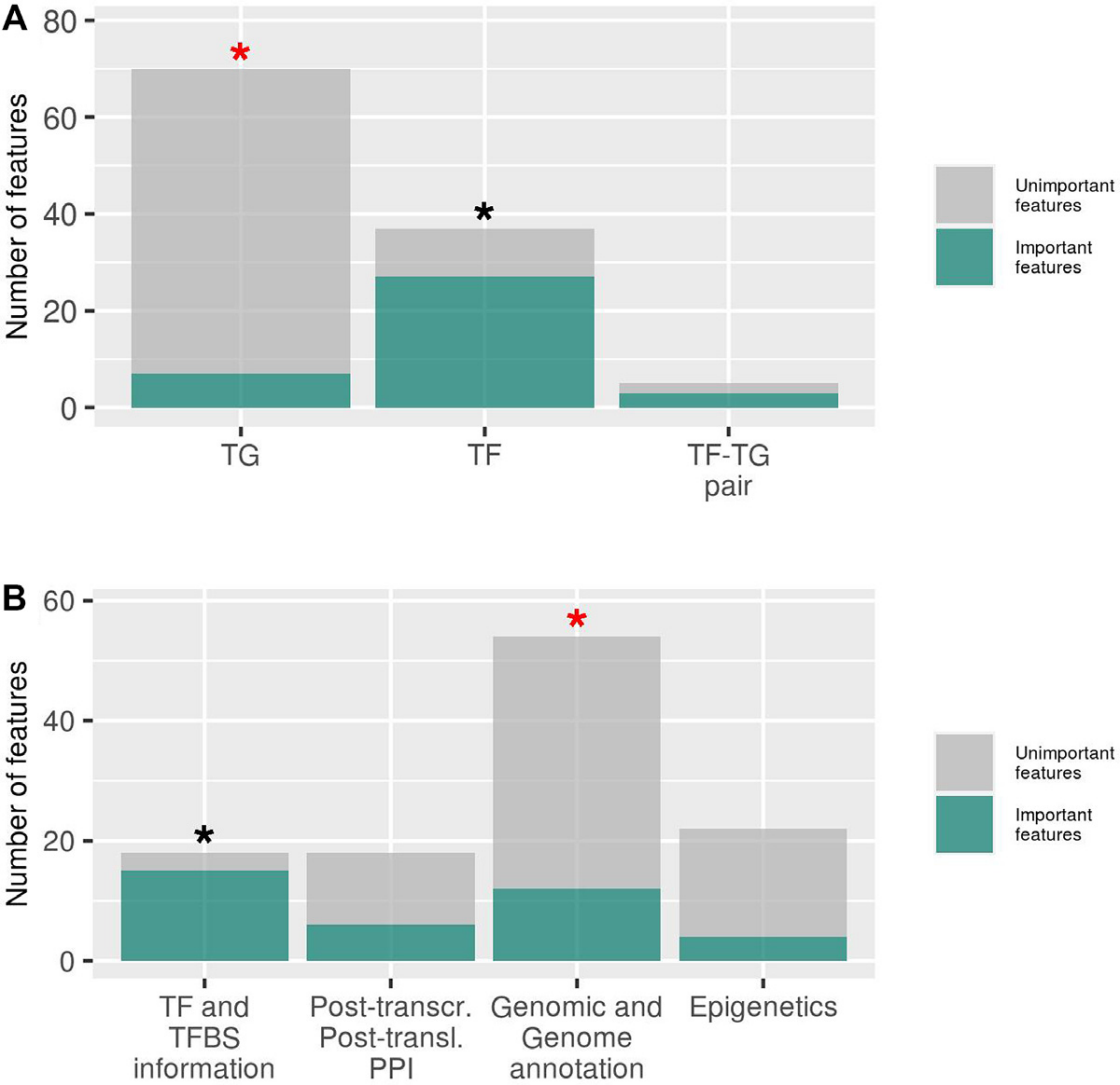
Number of features detected as relevant based on univariate testing or feature importance estimation from Random Forest models relative to all features associated with TG, TF and TF−TG regulatory pairs (**A**), and the four different feature categories (**B**). Asterisks represent statistical importance of enrichment (black color) or depletion (red color) of important features measured by Fisher exact tests with *P*-value < 0.05.

Interestingly, the identified discriminatory features (37 features) were significantly enriched for variables containing information about TFs (27 out of 37 important features, *P*-value = 5.3E−10, Fisher exact test; odds ratio = 16.91), and depleted for variables containing information about TGs (7 out of 37 important features; *P*-value = 2.4E−11, Fisher exact test; odds ratio = 0.046) (Figure 5A). The remaining informative variables concern the regulatory pair itself (three features). This suggests that by only using information about TFs, a reliable classification would perhaps be possible. Indeed, a Random Forest model, which uses as TF-related features only, performed well above random (median ROC−AUC = 0.67 and median PR−AUC = 0.14 in 20 times cross-validation), but worse than the model built using all variables (Supplementary Figure S2) (ROC−AUC, *P*-value = 1.2E−4; PR−AUC, *P*-value = 2.5E−5, Wilcoxon–Mann–Whitney test).

The union of all identified informative features (37 features) contains features belonging to all four considered categories (i) information about TFs and their TFBSs (15 features assigned as important out of 18 features from this category), (ii) post-transcriptional and post-translational regulation including protein−protein interactions (6 out of 18 features), (iii) genomic features and genome-derived annotation (12 out of 54 features), (iv) epigenetic features (4 out of 22 features), Figure 5B). The category representing information pertaining to TFs and their TFBSs (information about TFs and their TFBSs) was significantly enriched for informative features (*P*-value = 2.4E−6, Fisher exact test; odds ratio = 15.86). Depletion of the features assigned as important was observed in the category containing information on genomic features and genome-derived annotation (*P*-value = 0.027, Fisher exact test; odds ratio = 0.38).

From the set of selected features with high importance and a pronounced difference between correlated and uncorrelated pairs, the parameter with the highest impact on the Random Forest model, the largest MDA score, and also with the largest absolute effect size, was ‘Mean distance between TFBS of TF and TSS across all TGs’ (effect size = −0.42; MDA = 0.023). TFs with associated TFBS located, on average, closer to the TSS of its all TGs were more correlated in the expression, than those that bind more distant regions of promoter regions in general. Also when considering individual TF–TG pairs, those for which the respective TFBS is closer to the TSS were more correlated (‘Distance between TFBS and TSS in regulatory pair’: effect size = −0.29; MDA = 0.0036). In addition to the localization, the number of unique TFBSs in promoter regions was detected informative. TFs with a higher number of TFBSs in their promoter regions (‘Number of TFs regulating expression of TF’: effect size = 0.41; MDA = 0.009) and with a higher number of TGs (‘Number of targets assigned to TF (all targets and TFs)’: effect size = 0.10; MDA = 0.009) were enriched in the correlated TF−TG pairs. Those observations suggest that the localization of TFBSs in the promoter region as well as number of TGs of the TF can be associated with the expression regulation.

The inferred regulatory relationships between TFs and their TGs allowed us to also estimate the position of a TF in the network of regulatory interactions among TFs themselves (29). Conceptually, a TF-regulatory network can be partitioned into an initiation layer (top-level; no incoming, outgoing edges only), an intermediate processing layer, and an effector layer (bottom level; incoming, but no outgoing edges). We found that TFs positioned more at the bottom of the regulatory TF-TF network with more incoming than outgoing edges are associated with increased correlation; i.e. TFs that have as targets genes other than TFs, e.g. enzymes (parameter: ‘Number of TFs regulating the expression of TF over all regulatory connections of TF’, effect size = 0.089; MDA = 0.013).

In addition to the localization preferences of TFBSs and their numbers in promoter regions, the composition of TFBS-motifs was assigned as highly discriminatory in our model. TFBS-entropy (Equation 1) proved informative with larger entropies, i.e. less specific motifs, associated with uncorrelated pairs, while TFs binding to clearly defined motifs (low entropy) were found associated with correlated pairs (‘Mean entropy of DNA binding motif (TFBS) for five positions with lowest entropy of TF’: effect size = −0.36; MDA = 0.015; ‘Mean entropy of DNA binding motif (TFBS) of TF’: effect size = −0.32; MDA = 0.012), as were shorter TFBS motifs (‘Length of DNA binding motif (TFBS)’: effect size = −0.37; MDA = 0.010). In addition to the entropy and length of TFBSs, nucleotide composition of TFBS-motifs was found relevant for the correlation pattern of TF−TG pairs. TFs with a higher percentage of double versus triple H-bonded base pairs within their TFBS-motif (‘Ratio of AT to GC nucleotides in TFBS motif’: effect size = 0.27; MDA = 0.020) and with a lower ratio of purines versus pyrimidines (‘Ratio of AG to TC nucleotides in TFBS motif’: effect size = −0.13; MDA = 0.010) were associated with regulatory pairs with higher correlation. Therefore, TFs binding to more specific, shorter TFBSs and with a specific TFBS nucleotide composition showed higher correlation with their TGs.

Additional features with a high impact on the model or with a high effect size between correlated and uncorrelated TFs were associated with domain composition of the TF-protein. TFs with a higher numbers of DNA binding domains (DBD) (‘Number of DBD present in TF’: effect size = 0.16; MDA = 0.002) and with DBD localized further away from N-terminus of the protein (‘Start of first and second DBD in relation to N-terminus of TF protein’: effect size = 0.27 and 0.27; MDA: = 0.015 and 0.004, of first and second DBD, respectively) were enriched in the correlated pairs. This may, however, reflect TF-family specifics rather than having a molecular significance.

Processes involved in post-transcriptional and post-translational regulation also prove discriminatory with regard to correlated and uncorrelated regulatory pairs. TFs with a shorter theoretical path of protein–protein interactions (PPI) to establish an interaction with the largest Pol-II subunit (‘Distance to Pol-II in PPI network’: effect size = −0.33; MDA = 0.010) and with a higher number of interacting partners (‘Node degree in PPI network of TF’: effect size = 0.05; MDA = 0.010) were found enriched in the correlated TF−TG pairs. At the level of mRNAs, TFs with a high numbers of interacting miRNA (‘Number of target sites of miRNA in mRNA of TF’: effect size = 0.20; MDA = 0.004) and also with a higher similarity with mimic miRNA (‘Maximum of free energy ratio of mimic miRNA:mRNA duplex of TF’: effect size = 0.27; MDA = 0.004) were found overrepresented in highly correlated features. Those results are in line with predictions that post-translational and PPIs can influence the expression correlation of regulatory pair members.

With regard to TGs, while no molecular features associated with TGs or their promoters met the importance threshold in the feature extraction based on RF models, some of these features were selected as important by univariate testing. Evolutionarily younger TGs exhibit increased correlation to their respective regulating TF compared to evolutionarily old TGs (‘Evolutionary age of TG’: effect size = 0.26; MDA = 0.005). Interestingly, while the evolutionary age of the TF was not informative at all (Supplementary Figure S5 and Table S4), the difference of evolutionary age was found relevant by univariate testing, with increasing age differences (younger TGs paired with older TFs) being associated with increased correlation (‘Difference between evolutionary age of TG and TF’: effect size = 0.18, MDA = 0.005). TGs which are a part of correlated pairs show differences in the promoter regions in regard to specific motifs and also nucleotide composition. TGs with present TATA-box motif in their promoter (‘Presence of TATA-box motif in promoter of TG’: effect size = 0.19; MDA = 0.0004) and with a lower percentage of GC and CG di-nucleotides (‘Percentage of GC in promoter sequence of TG’: effect size = −0.15; MDA = 0.001; ‘Percentage of CG in promoter sequence of TG’: effect size = −0.16; MDA = 0.001) in the promoter region were found to be associated with higher correlation. Differences between TGs from correlated and uncorrelated pairs were also observed at the level of epigenetics. TGs with a lower number of differentially methylated DNA regions in the gene body (‘Number of differentially methylated CpG in gene body of TG (52,53)': effect size = −0.23 and −0.16; MDA = 0.001 and 0.0004, respectively) were found enriched in the correlated pairs. Interestingly, TFBSs located in stable DHS regions in the promoter were characteristic for the correlated regulatory pairs (‘TFBS of regulating TF is located in stable DHS’: effect size = 0.19; MDA = 0.001) along with a higher number of dynamic DHS located in a promoter region of TG (‘Number of dynamic DHS in promoter of TG’: effect size = 0.18; MDA = 0.001).

### Feature interaction effects

The power of applying the RF classification methodology does not only lie in its potential to identify features with more complex characteristics, but also to discern variable interactions. As a metric for gauging feature interaction, we applied the *mean conditional depth* between two variables as described in (62). See Materials and Methods, for more information and explanation of rationale. Both, the frequency of variable pairs being picked jointly as well as their mean conditional depth between them in the RF classification trees reflect on variable interactions.

**Figure 6.**
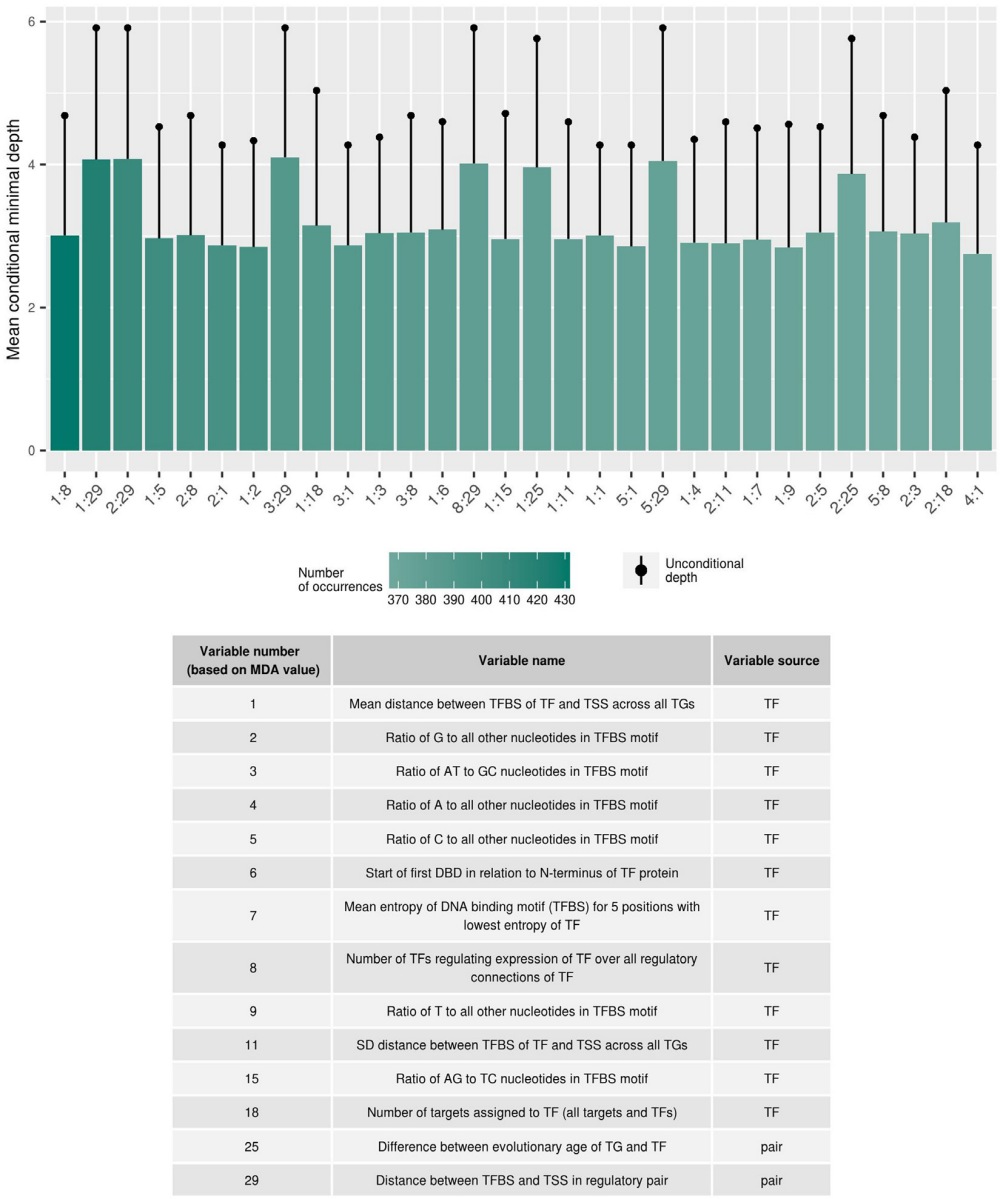
Feature interactions with most frequent occurrences in the Random Forest classification run between correlated and uncorrelated pairs. Height of the bar represents the mean conditional depth between a variable considered conditioning (first variable index in the colon-separated pair) and a second variable (second index) tested for interaction effects. Indices assigned to each variable, listed in the table below the plot, correspond to the name of each feature (or cluster of features for correlated features) sorted by decreasing importance as judged by MDA obtained from the Random Forest model. Color of the bar represents the number of occurrences of each pair in the Random Forest models, counted as the number of trees containing this interaction. Black dots represent the unconditional mean depth of the second variable of the colon-separated variable pair in the Random Forest models. All values are averages over 20 cross-validations. Smaller values of the mean conditional depth with associated higher unconditional depth as well as increased occurrences indicate interaction effects. See Materials and Methods for more details. Source indicates to which component of the TF–TG the respective variable is associated with (TF, TG or TF–TG pair)

The 30 most frequent interactions between features present in the RF model were observed between only 14 features out of the 29 features with the largest MDA score (Figure 6). Interestingly, almost half (14 interactions) of the 30 most frequent variable interactions were found with the ‘Mean distance between TFBS of TF and TSS across all TGs’ being the conditioning variable, and five observed interactions were found associated with the feature ‘Distance between TFBS and TSS in regulatory pair’ as the variable conditioned on other features such as features associated with TFBS motif composition and average distance distribution between TFBS and TSS among all TGs. This suggests the distance of TFBSs to TSSs is highly relevant with regard to conditional interactions with other features. Surprisingly, no interaction between features that concern a TF and its associated TG were detected among the top-30 variable pairs.

Taken together, aside from identifying particular TF-families that were associated with elevated levels of co-transcriptional coupling, we discerned a set of biological and molecular characteristics that distinguish highly correlated from uncorrelated TF–TG pairs. The majority of them were associated with TFs, but also with the status of the epigenetic modifications of the promoter of target genes and their evolutionary age.

## DISCUSSION

Correlation of expression of transcription factors (TFs) and their target genes (TGs) is commonly thought to be a hallmark feature by which regulatory relationships between them become evident. However, when probing for correlated gene expression of known regulatory TF–TG pairs, only a weak signal of increased correlation levels, setting true from random pairs apart, has generally been observed - also in this study. Using data available for the plant *Arabidopsis thaliana*, we investigated whether this absence of a clear-cut signal is generally true for all TF–TG pairs, or whether there are molecular determinants that render specific TF–TG pairs correlated at the transcriptional level, while the regulation of others is more complex such that it is not evident from expression data alone. Indeed, we found that particular TF-families exhibit pronounced transcriptional coupling with their TGs (WRKY, TCP, MYB, HB, E2F-DP, NAC; Table 1). As this tendency of some TF-families to be more directly transcriptionally coupled to their TGs they must have underlying molecular determinants, we set out to test a wide range of molecular parameters that may influence the degree of transcriptional coupling and which capture different levels of gene expression regulation (information on TF and their TFBS, post-transcriptional and post-translational regulation including protein–protein interaction, genome and genome annotation derived information, and epigenetics). As a result, our analysis revealed a number of molecular features that appear associated with increased or decreased levels of transcriptional coupling. Hence, when aiming to infer regulatory relationships from the expression data, considering the detected features as part of the inference strategy appears very promising. And with this study, we believe to have laid the foundation for such attempts.

We focused our analysis on the pairwise relationship between TFs and their TGs. However, it is known that TGs are often, if not generally (72–76), regulated by the joint action of several TFs. Hence, treating expression correlation as a pairwise phenomenon may seem inappropriate. However, our rationale was to identify simple and direct TF–TG relationships as evidenced by transcriptional coupling as well as departures from this simple model, revealed by uncorrelated expression. Conceptually, the simple model would assume the transcription of a TF to always indicate the transcriptional regulation of its TGs, irrespective of additional factors. By contrast, our reasoning assumes more complex relationships to be associated with de-coupled, and thus uncorrelated expression. With the choice of many diverse molecular features, including, for example, the joint action of several TFs (e.g. features ‘number of TF-binding sites in promoter’, ‘node degree in PPI network of TF’), we intended to cover a broad spectrum of potential molecular determinants of either simple expression coupling or more complex regulations.

### Transcriptional coupling is associated with stress response

Before discussing the found molecular determinants of correlation coupling in more detail, it is worthwhile to ask whether there are some general trends associated with TFs and their TGs in the correlated pairs. First, we observed an enrichment of six TF-families in the correlated pairs and of 13 TF-families in the uncorrelated regulatory pairs. Based on the functional annotations of the six transcription TF-families enriched in the correlated set (77), they are involved in a broad range of diverse biological processes, including stress response and developmental or cell-regulatory processes. For example, the TF-families NAC, WRKY, MYB-TFs have been reported to regulate senescence (78,79). TCP–TFs have been shown to regulate flower-developmental programs (80), and E2F-DP-TFs cell-cycle processes (81). Members of the MYB, TCP and WRKY have also been reported to take part in the abiotic stress and biotic stress response (80,82,83) of the TF-families enriched in the uncorrelated TF–TG pairs, C2C2-Dof and BBR-BPC have been reported to regulate the differentiation of tissues and regulation of the seed and flower development (84,85). Members of bZIP and AP2-EREBP families are involved in abiotic stress and developmental processes, e.g. flower and seed development as well as seed germination and early senescence (79,86,87). Thus, based on the reported functional involvement of the enriched TF-families in correlated and uncorrelated TF–TG pairs, a separation into distinct biological processes, such as stress response versus developmental processes, seems unclear. However, when tested statistically and basing it on TF–TG pairs rather than TF-families, there does seem to be a clear separation of GO-process annotations in the two sets (correlated vs. uncorrelated) nonetheless. Correlated TF–TG pairs seem frequently associated with stress-responses, i.e. responses to external cues, while uncorrelated pairs seem to associate more with developmental programs (Table 2). This may suggest that the functional differences may not be tied to TF-families as a whole, but to the diverse properties of their respective members. In addition, when molecular features were analyzed for importance in the correlated TF–TG set, features associated with multi stimuli response processes were detected enriched, e.g. presence of TATA-box in the promoter of TGs (18), and enrichment in the evolutionarily younger genes, which are enriched in stress response GO categories (Supplementary Table S5). While this may to some degree be a consequence of the available expression data, in which stress-experience conditions dominate, this may be interpreted as a difference in programs (stress vs. developmental) and their regulation.

### Molecular features affecting transcriptional coupling of TFs and their TGs

The observed general trends in the correlated TF–TG pairs with regard to enrichment of certain TF-families and association with stress response processes, should be supported by molecular features, which can influence the expression correlation between TFs and their TGs. When the set of molecular features considered in this study was analyzed, a strong enrichment of features associated with TFs was observed (27 out of 37) (Figures 4 and 5A). In line with the TF-family enrichment analysis, a high importance was assigned to features associated with protein domain structure annotation and TFBS composition. A significant difference was observed between correlated and uncorrelated TFs with regard to the position of the DNA binding domain (DBD) in relation to the N-terminus of the TF protein. TFs with a DBD located further away from the N-terminus were observed to be more correlated with their TGs. TFs with a more specific binding motif (lower entropy of the TFBS motif) show a higher correlation with their TGs, and TFs with characteristic nucleotide compositions of their TFBS (higher ratio of AT over GC pairs (double versus triple H-bonded base pairs)), were found enriched in correlated pairs. As the classification of TFs into different TF-families is performed based on the domain architecture of the TFs, it is possible that the importance of localization of the DBD in the protein sequence may be an artificial effect associated with the architecture of certain TF-families rather than a reflection of a 3D-structural consequence of different relative DBD-sequence positions on the regulatory mechanisms. Nevertheless, earlier studies reported a significant association of the relative position of DBDs along a TF’s primary protein sequence and the activity of a TF to function as activators or repressors (88,89). By contrast, features associated with TFBS motif composition may indeed reflect molecular determinants of the higher correlation between certain TFs and their TGs. More precise TFBS motifs suggest a more specific set of TGs, while the lower ratio of the CG to AT pairs in the targeted motif may be associated with a lower energy needed to dissociate the two DNA helix strands (90), which is supported also by our observations that a low content of the CG and GC di-nucleotides was detected in promoter regions of correlated TGs.

Correlated TF–TG pairs were found to be associated with a shorter theoretical interaction path needed to establish an interaction between TFs and the largest Pol-II subunit. This observation is in a line with the rationale that a smaller number of interactions is less dependent on additional factors, which could interfere with the correlation between the TF and its TGs.

TFs that are highly correlated with their TGs are regulated themselves by a larger number of TFs, in comparison to TFs not generally found in correlated pairs. This, along with the observation that TFs in correlated pairs have relatively fewer out-going regulatory interactions (‘Number of TFs regulating expression of TF over all regulatory connections of TF’) suggests that correlated TFs are closer to the effector layer rather than perception and processing layer of regulatory interactions (29).

The observed shorter half-lives of mRNA molecules of highly correlated TFs suggest a relevance of degradation pathways affecting mRNA level, which appears plausible as clearance (along with induction) can be seen as a requirement for TF–TG correlation. Degradation may be linked to miRNA activity via miRNA-target-mRNA-cleavage. Indeed, the mRNA of highly correlated TFs is targeted by a larger number of miRNAs compared to uncorrelated TFs, although a lower degree of the sequence complementary (measured by the ratio of the minimum free energy between predicted miRNA-mRNA to perfect complementarity) was observed in the correlated TFs. This suggests that not only the mRNA cleavage pathway, which is associated with high complementary miRNA-mRNA binding (67), is involved in the regulation of the mRNA level, but also the translation repression via miRNA interaction that may influence activity of the mRNA of TFs.

Surprisingly, we observed an underrepresentation of important features associated with correlated regulatory pairs that describe properties of TGs (Figures 4 and 5A). Nevertheless, the TG-features that were found relevant, revealed interesting properties of the highly correlated pairs. Evolutionarily younger TGs and TGs regulated by older TFs were more likely expression correlated. This implies that as new genes emerge in evolution, they are initially under the transcriptional control of TFs, with more complex regulatory relationships developing later and over evolutionary time frames. This is in line with observations that in prokaryotic systems; i.e. evolutionarily old systems, such as *E. coli*, evidence for transcriptional coupling has been stronger (13), establishing it as an initial mode of regulation. The higher correlation observed in younger TGs could be also associated with a higher dynamics of expression in younger genes. Dynamic changes in the expression of the younger genes were observed not only during embryogenesis (48), but also in our expression dataset. By contrast, older genes exhibit more constant (and higher) gene expression across the profiled conditions (Supplementary Figure S7). Also, we observed that younger genes are enriched for stress response and signaling process involvement, while older genes seem more associated with housekeeping functions (Supplementary Table S5). While the stress and signalling processes can be expected to be dynamic and condition-specific, housekeeping functions can be assumed with more constant corresponding expression levels. As more constant dependent variables (TGs), by definition, are correlated less to their respective independent cause (TF), the difference with regard to evolutionary age can be explained via the respective processes involvement and associated dynamic behavior.

Analyzing the localization of TFBSs in promoters of TGs, we observed that a localization of the TFBS of a particular TF in regions with stable open chromatin marks was more likely to result in increased correlation with its target gene (feature ‘TFBS of regulating TF is located in stable DHS’). This supports the notion that the regulation of the promoter sequence by the chromatin status influences the expression correlation. If the TFBS is not localized in stably open chromatin, then an additional layer of expression regulation, via the localization of nucleosomes, could perturb the expression correlation within a regulatory pair. This observation is in line with the reported increased accuracy of GRN inference methods that combine information of open chromatin and TFBS localization (91).

In addition to the localization of the TFBS in the stable open chromatin, the localization of TFBSs in the promoter of the TG in general i.e. distance between TFBS and TSS, for each regulatory pair and also as a general feature of the TFs (averaged over all TGs of a given TF). Correlated TFs bind, on average, closer to the TSS of their TGs than uncorrelated TFs. This pattern is also observed when we tested the standard deviation (SD) of the average distance between TFBS and TSS for enriched TF-families. TFs that are part of the correlated TF-families have narrower binding localization intervals (SD of the average distance between TFBS and all TGs = 78.0 bp) in comparison to the uncorrelated TF-families (SD of the average distance between TFBS and all TGs = 140.9bp) (*P*-value = 2.9E–5, Wilcoxon–Mann–Whitney test). This observation is also reflected in the observed feature interactions (Figure 6). The distance between TFBS and TSS in each regulatory pair was often conditioned on features associated with TFBS composition. This suggests that for a group of TFs, characterized by nucleotide motif composition of their TFBS and possibly reflecting assignment to a particular TF-family, the distance between TFBS to the TSS of TGs plays an important role in regulation of the expression.

Of note in particular with regard to considering additionally acting TFs and our rationale to focus on pairwise TF–TG relationships: we found that increased number of PPI seems associated with elevated TF–TG correlation levels (Figure 4). Also when considering interactions at the TF-family level, the number of cross-TF-family interactions as reported in (92) for correlated TF-families (Table 1) was, on average, larger (interaction with 12.8 different TF-families) than for uncorrelated TF-families (on average, interacting with 6.8). Both these observations suggest that transcriptional coupling may not be associated with few, but rather with more interactions.

In selecting molecular features, each one was chosen with a specific rationale, rendering it a likely candidate to influence the strength of transcriptional coupling. Therefore, it is also interesting to review those features that we thought were relevant, but were not reported as part of the set of most relevant features (Figure 4). Of note, in particular, ‘number of phosphorylation sites of TF’, representing an important post-translational modification, was not among the most informative features. Phosphorylation of TFs has been shown to activate TFs (14,68). Hence, phosphorylation may render constantly expressed TFs condition-specifically active, and therefore, would be expected to be increased in the set of uncorrelated TF–TG pairs at the transcript level. Indeed, in line with expectation, while not reported among the most important features, uncorrelated TF–TG pairs have more phosphorylation sites than correlated pairs (effect size = –0.06, MDA = 0.005, Supplementary Figure S4 and Table S4), yet the adjusted *P*-value was above the level of significance, p_FDR_ = 1.5E–6.

#### Promoters vs. enhancers

We based the pairing of TFs and their TGs on observed binding of the respective TFs to the 500 bp region upstream of the transcription start site (TSS), considered the gene promoter. TFs are also known to bind to distant regions relative to the gene they regulate, so-called enhancers (93,94). Also, TFs binding to intragenic and intronic regions have been reported to have regulatoratory effects (95). In our study, such distant and intragenic regulatory binding would not be considered. If, as reported (96) enhancers exert the regulatory effect over large genomic distances, also in *A. thaliana*, correct TF–TG pairing is challenging such that a pairing based on some sequence-interval would be highly unreliable. However, it was recently suggested that enhancer action in *A. thaliana* is rather local (96). Hence, and because the 500 bp extends beyond what is considered the core-promoter (70 bp around TSS (97)) our study may, in fact, capture enhancer sites to an appreciable degree already.

### Technical aspects, limitations

#### DAP-seq data

We took as the dataset of true regulatory TF–TG interactions data generated by applying the DNA-Affinity-Purification-Sequencing (DAP-Seq) protocol (17,98). In this assay, binding of a set of TFs to genomic DNA was detected. As the presented genomic DNA in this assay is naked (i.e. without any coating by proteins or other factors, except DNA methylation, which is captured in the PCD) and fragmented (i.e. without long-range 3D structural interactions), the assay results cannot be expected to directly capture the *in vivo* binding of TFs. Nevertheless, when results of ChIP-seq were compared with DAP-seq, significant overlaps between the two techniques were observed (36–81% of peaks present in ChIP-seq were also present in the DAP-seq) (17). We can assume that those regions that are not present in the DAP-seq set may need additional co-binding factors or specific chromatin states not present in the DAP-seq experiment (e.g. pioneer TFs binding to nucleosomes). This shows that the DAP-seq dataset may indeed contain some false-negative regions of TF binding events. On the other hand, not all peaks reported by DAP-seq are reported in the ChIP-seq assay either (17). As mentioned, TF binding event is highly dependent on the structure of chromatin, e.g. openness state, which can vary between conditions. Furthermore, and as the developers of the DAP-seq protocol noted, the set of amenable TFs showed a bias to particular TF-family (bZIP, NAC, WRKY), while others were underrepresented (MADS, C3H) (17). Moreover, only one TF at a time was tested for the DNA binding, which eliminates all potential binding events, where the co-binding factor is necessary for establishing the DNA binding (false negatives). Hence, our results have to be seen in the light of these limitations. In particular, the over/ underrepresentation of particular TF-families limits the general validity of the relative frequency of correlation characteristics (occurrence of correlated vs. uncorrelated TF-target-gene expression). By contrast, the limitations ‘naked’ and ‘fragmented’ may not impact negatively on our analyses. We required binding sites to lie within gene promoters, a requirement to reduce the number of false-positive TFBS assignments and furthermore, the missing properties, such as the presence of additional factors (histone binding etc.) or the influence of varying accessibility (DHS-sites) were added by us as features, allowing us to gauge their importance on the transcriptional coupling of TFs and their TGs.

With regard to the detected low entropy of TFBSs associated with TFs displaying increased TF–TG correlation, it is of note that the position-weight-matrices (PWM) of the TFBSs (TFBS-motifs) were computed from the DAP-seq binding events themselves. Thus, increased TFBS-entropy may also reflect the inclusion of false-positive binding events, which, when included, introduce false TF–TG pairs.

#### Expression dataset

We used a large expression dataset generated using microarray experiments, which has the advantage that a very large and broad set of experimental conditions (5,296 hybridizations) was screened. As in recent years, RNA-seq-based methods have largely replaced microarray-based expression profiling, we also tested whether our results hold when basing it on available RNA-seq data. Indeed, expression correlation of TF–TG-pairs was consistent in both datasets, microarray and RNA-seq data (Pearson correlation coefficient *r* = 0.49; Supplementary Figure S1). Furthermore, we obtained high correlation between determined feature importance values, measured by MDA, of models based on the microarray and RNA-seq expression dataset (Pearson correlation coefficient *r* = 0.84), and a large overlap between features selected as important between two models was observed (21 features out of 23 features selected as informative for both models (microarray and RNA-seq-based). Hence, we believe that our results are valid irrespective of the underlying expression profiling technique.

#### Expression correlation metrics

We used Pearson correlation to detect correlated expression. Pearson correlation is best suitable for normally distributed data exhibiting linear relationships. While other metrics to detect more complex and non-linear relationships such as mutual information (99) have been proposed, we intentionally employed a metric that detects simple relationships, as we wished to extract those, in which an increase of TF-expression is associated with an increase of expression of its TG, likewise a decrease in TF with decrease of TG-expression. As long as this relationship is monotonic, Pearson correlation will identify them with reasonable sensitivity. With regard to actual threshold of detecting a pair as correlated (*r* > 0.4 in this study), we tested other thresholds as well, yielding qualitatively similar results, but with a trend that more pronounced effects were obtained, when the contrast between correlated and uncorrelated pairs was increased, i.e. requiring larger correlation coefficients for TF–TG pairs to be considered correlated, which has to be balanced with the simultaneous decrease of observations.

#### Time-shifted correlation

In the expression correlation analysis, we computed correlation coefficients based on values for TFs and their TGs paired up in the same sample. This means that possible time delays between TF and the expression of their TGs, with TFs assumed to precede any changes of expression of their TGs, were not taken into account. While considering such delays (time-shifted correlation) was shown to be informative (100), we did not consider it here, as most of the microarray experiments, on which our analysis was based, were not time-series data, but case-control studies such that time-shifted correlation cannot be applied. Furthermore, it is uncertain what the relevant time delay interval should be (100). In essence, we focus on those TFs and their TGs, which show synchronous expression change given the temporal resolution of available experiments.

#### Positively vs. negatively correlated TF–TG pairs, activator vs. repressor TFs

Transcription factors are known to act as activators or repressors, with some TFs acting as both activators and repressors depending on the condition or co-factors (66,78). Intuitively, activator TFs should coincide with positively correlated TF–TG pairs, as the expression of a TG regulated by an activator TF should increase along with an increased expression of the regulating TFs. By contrast, when TFs repress the expression of their TGs, a decrease of the TG should be observed in response to increased TF-expression, and hence repressor-TF and their TGs should be negatively correlated.

In this study, we considered as regulatory and transcriptionally coupled pairs only those, in which the expression of the TF is positively correlated with TGs. Therefore, and following the intuition outlined above, our results may seem to relate to TFs acting as activators only. However, upon further scrutiny, this conclusion may be incorrect and the equivalence of correlation and function may be more intricate. First, we did not observe increased frequencies of significant negative regulation of true TF–TG pairs compared to random TF–TG pairs, whereas significantly more than expected positive correlations were indeed evident (Figure 1A and C). This absence of an expression signal originating from TFs functioning as repressors is in agreement with reports in *E. coli* that surprisingly, repressor TFs are positively, rather than negatively correlated with their TGs when probed across different conditions, but at a single timepoint (13). When tested in our dataset and based on published results on TFs to function as activators or repressors (101), we confirm this finding to hold in Arabidopsis as well. Repressor TFs are also associated with positive expression correlation to their known TGs (Supplementary Figure S8). We believe, the solution of this apparent contradiction relative to intuition and definition of what we consider repressor function must lie in the consideration of time-shifted effects as discussed above. However, this surprising result also means that our results hold true for repressor TFs as well, and we suggest to interpret our study more generally as studying regulatory interactions, without qualifying them as activation or repression.

Of further note, it has been reported also in *E. coli* that activating TFs bind DNA upstream of their target genes only, while repressors bind downstream or as a combination of upstream and downstream binding events (28). Assuming a transferability of these findings to eukaryotic systems, by identifying TF–TG pairs via upstream promoter locations of TFBS only, our dataset can be considered enriched for activator TFs.

The primary goal of this study was to contrast correlated and non-correlated TF–TG pairs. To check whether subsetting on positive correlation only does not introduce a contrast of positive versus negative correlation in addition, we re-run parts of our analyses for true pairs considered with |*r*| > 0.4, i.e. including both positively and negatively correlated pairs. The obtained results were virtually identical as reported for *r* > 0.4. The TF-families found enriched (Table 1, Supplementary Table S6) were highly correlated (Spearman correlation of –log(*P*)-enrichment: 0.93, *P*-value = 3.4E–06; as well as the determined feature importance values (MDA, Pearson, *r* = 0.99; *P*-value = 7.4E–99)). Hence, our results do not seem to be tainted by an additional qualitative difference.

#### Considered features

We considered a wide array of 135 different molecular parameters as determinants of transcriptional TF–TG coupling (Supplementary Table S2). It is, however, possible that important factors were missed, either by being unaware of them or that insufficient data was available. Data availability was, in particular, a limitation with regard to epigenetic factors, where a broader profiling of epigenetic marks and properties, and associated with the experimental conditions, for which expression data is available, would have been desirable. Of note, we intentionally left out all parameters concerning actual expression levels and their variance of TFs and TGs. When including those parameters, they proved to be very informative (not shown), which, however, is very likely simply a statistical artifact that correlation is more likely to occur when the correlated variables display some dynamic range relative to the noise level of expression and the measurement thereof. Also, expression levels cannot be considered molecular determinants by themselves, and rather are a consequence than the cause.

The correct identification of gene promoter regions as genomic sequence intervals around (here, strictly upstream) of the transcription start site (TSS) of genes, evidently, is of central importance for our approach. Here, we wish to point out that we assumed a single TSS per gene. However, it is known that eukaryotic genes, in general, as well as Arabidopsis genes, in particular, may have several TSSs per gene (102), with possibly associated individual promoter regions. However, the primary expression data used in this study (microarray-based) does not allow for isoform-detection, in particular not the detection of different TSSs. Thus, our study has to be interpreted with this limitation in mind.

#### Feature extraction

We pursued two strategies to identify relevant features, univariate statistics and machine learning based on Random Forest. While the former allows gauging the influence of all individual features and probes for simple binary class differences, the latter allows for more complex, non-linear relationships as well as testing for interactions (Figure 6). We wish to stress that the goal of implementing the machine learning methodology was not to develop a classifier as such, but rather to use it as a feature selection engine, which at the same time also allows for assessing the predictability of outcome, and hence, relevance of the considered features.

## CONCLUSIONS

The generally observed weak transcriptional coupling between transcription factors (TFs) and their target genes (TGs) is not a reflection of a general weak association at the transcriptional level. Instead, molecular determinants render particular TFs more transcriptionally coupled with their TGs than others. The elucidation of these factors, as reported here for the plant *Arabidopsis thaliana*, may contribute to a deeper understanding of gene expression regulation.

## Supplementary Material

gkaa927_Supplemental_Files
