# Supplementary material for: Determinants of correlated expression of transcription factors and their target genes
Source: Nucleic Acids Res. 2020 Oct 26;48(20):11347–69. doi: 10.1093/nar/gkaa927 (PMC7672440; doi:10.1093/nar/gkaa927)
Supplement: gkaa927_Supplemental_Files [file gkaa927_supplemental_files.zip › Supplementary materials.pdf]

Supplementary material

Determinants of correlated expression of transcription factors and their target genes.

Adam B. Zaborowski and Dirk Walther

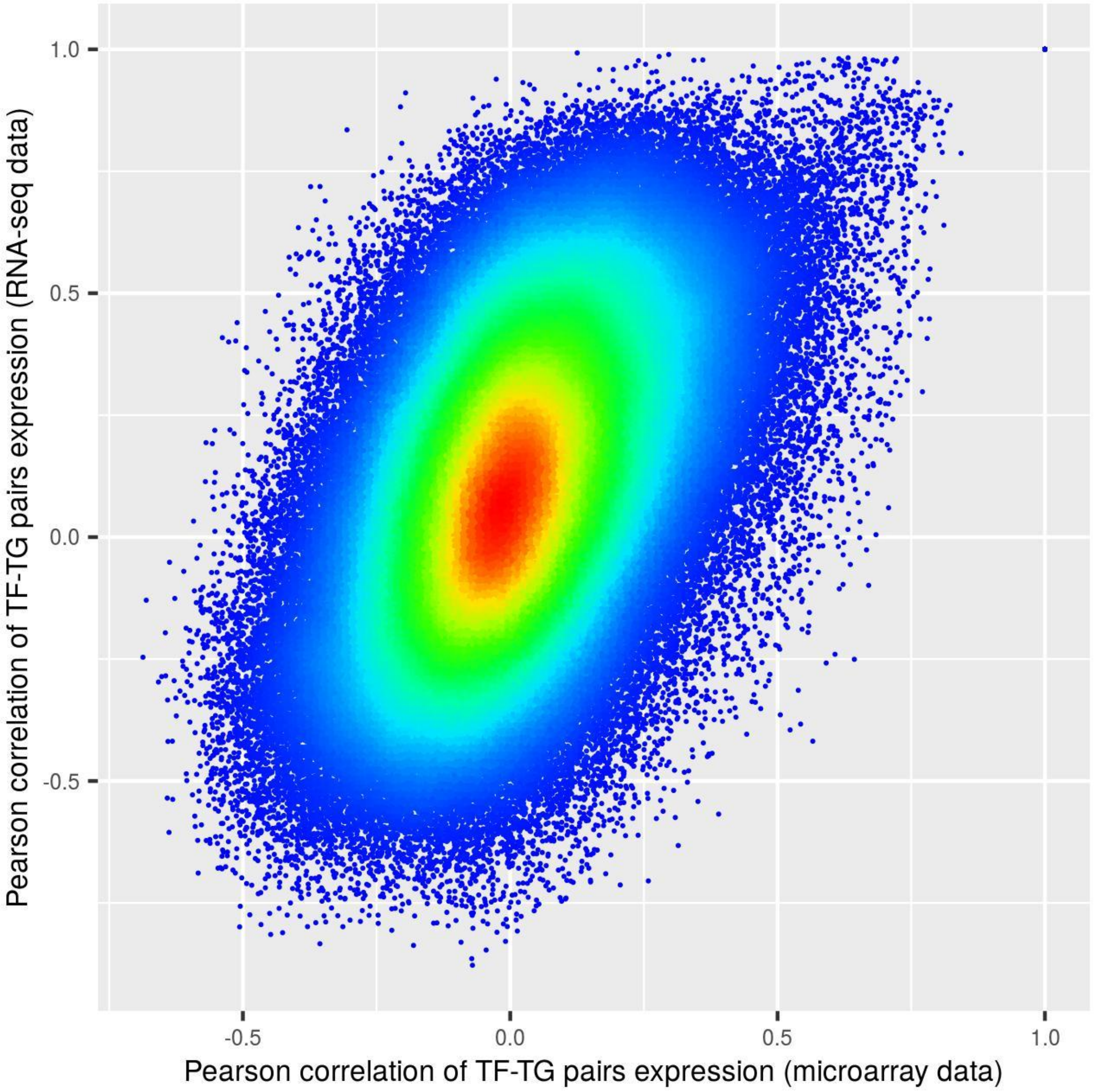

Supplementary Figure S1. Comparison of the Pearson correlation coefficients of TF-TG pairs expression measured by microarray hybridizations or RNA-seq data. Pearson correlation between Pearson correlation coefficients from two expression sets was equal to 0.49 (p-value < 2.2E-16). Color-scale indicates the density of points from blue (low) to red (high).

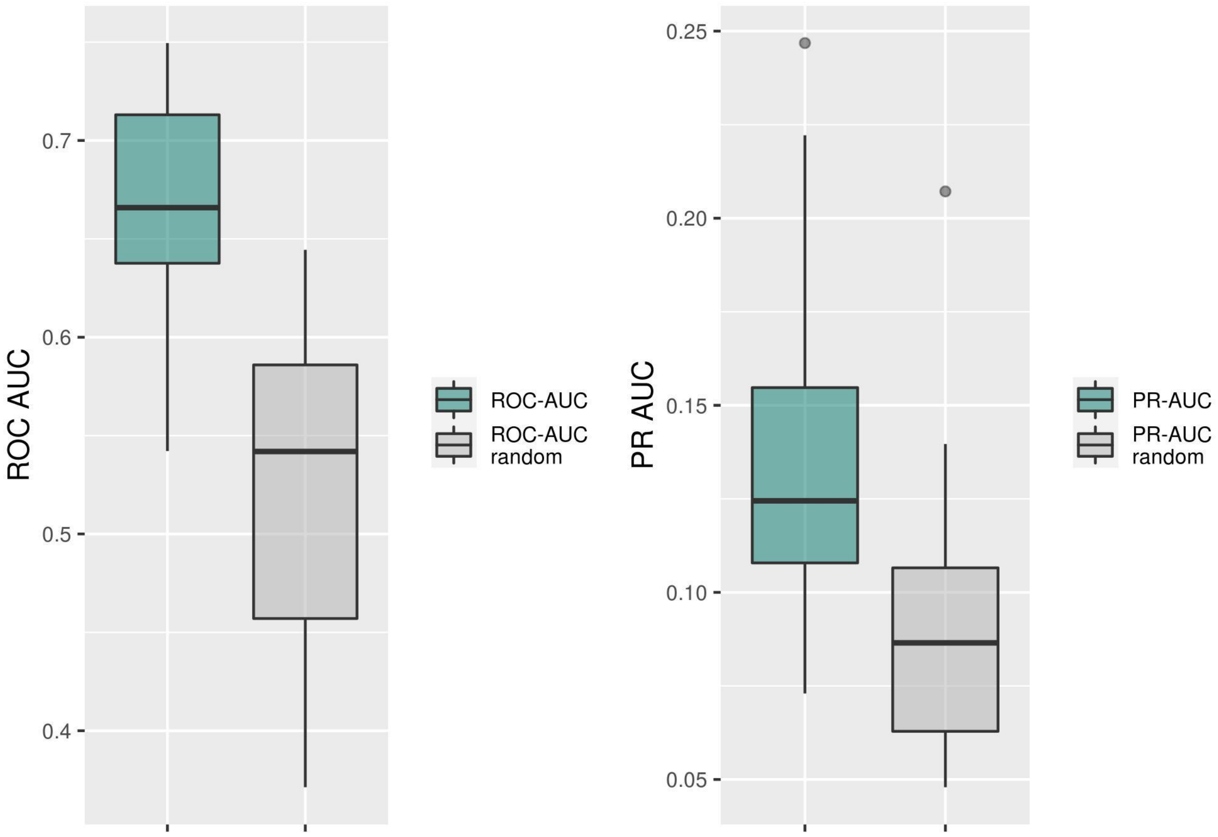

Supplementary Figure S2. Performance of the Random Forest classification of TF-TG pairs into the correlated or uncorrelated set based on the collected features associated with TFs only, measured by AUC of the ROC and PR curve. Green boxes represent the distribution of AUC values for prediction of the Random Forest model and grey boxes represent the distribution of AUC values for permuted class-labels. Values obtained from 20 times cross-validation runs. Values of ROC-AUC and PR-AUC obtained for our model were significantly higher than values obtained for permuted class-labels. Significance of difference was observed with Wilcoxon–Mann–Whitney test p-value = 5.8E-11 and 1.1E-7, respectively.

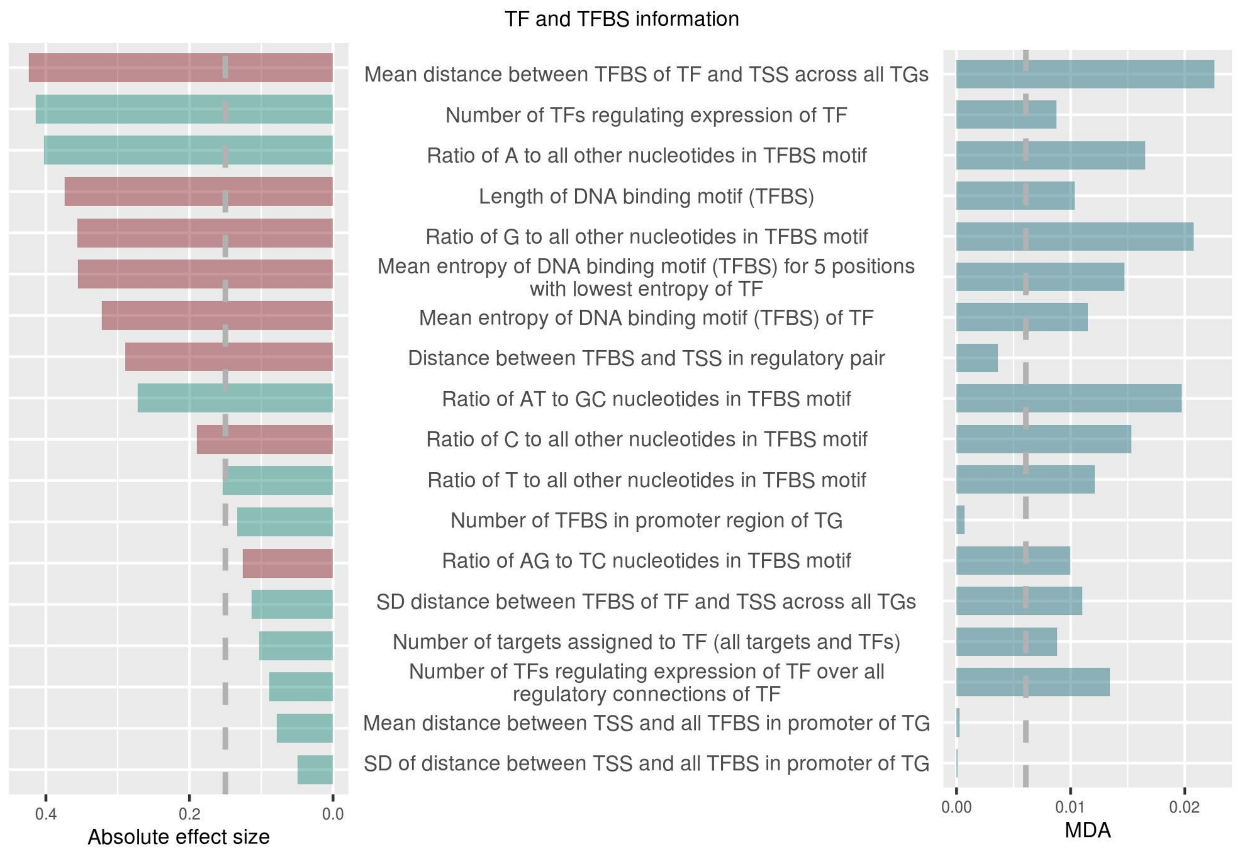

Supplementary Figure S3. Feature effect sizes and importance in the Random Forest classifier distinguishing between correlated and uncorrelated pairs for feature class 'TF and TFBS information'. The left panel presents the absolute effect size (Cohen's D) between correlated and uncorrelated regulatory pairs. Features with green/red bars show higher/lower values in correlated compared to uncorrelated pairs. Right panel presents Mean Decrease of Accuracy (MDA) that results from a permutation of the selected feature in the Random Forest after training, but before prediction. Vertical dashed lines present arbitrarily chosen thresholds for absolute effect size (0.15) and for MDA (0.0061), used to identify the important features. TF-transcription factor, TG-target gene, SD - standard deviation.

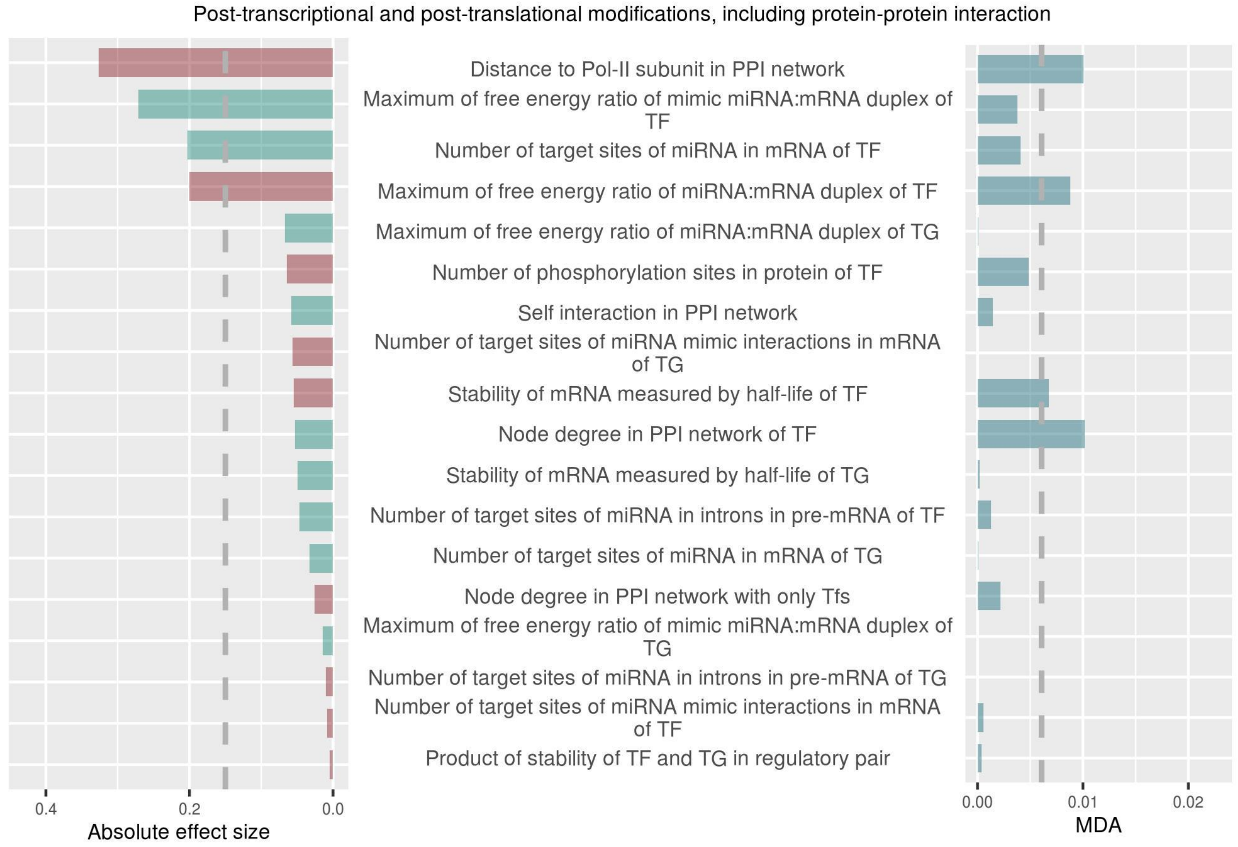

Supplementary Figure S4. Feature effect sizes and importance in the Random Forest classifier distinguishing between correlated and uncorrelated pairs for feature class 'Post-transcriptional and post-translational modifications, including protein-protein interaction'. For additional information, see legend of Supplementary Figure S3.

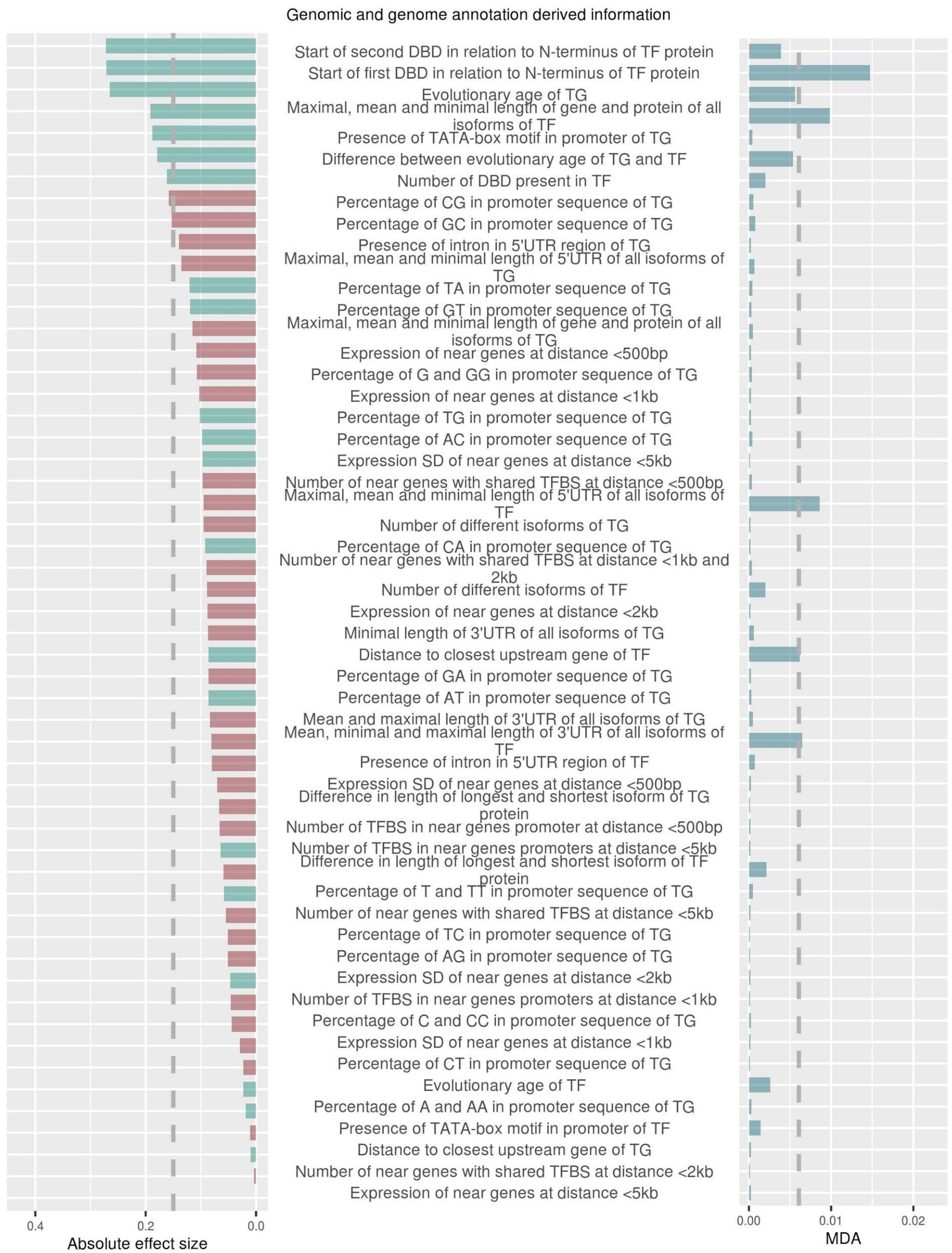

Supplementary Figure S5. Feature effect sizes and importance in the Random Forest classifier distinguishing between correlated and uncorrelated pairs for the feature class 'Genomic and genome annotation derived information'. For additional information, see legend of Supplementary Figure S3.

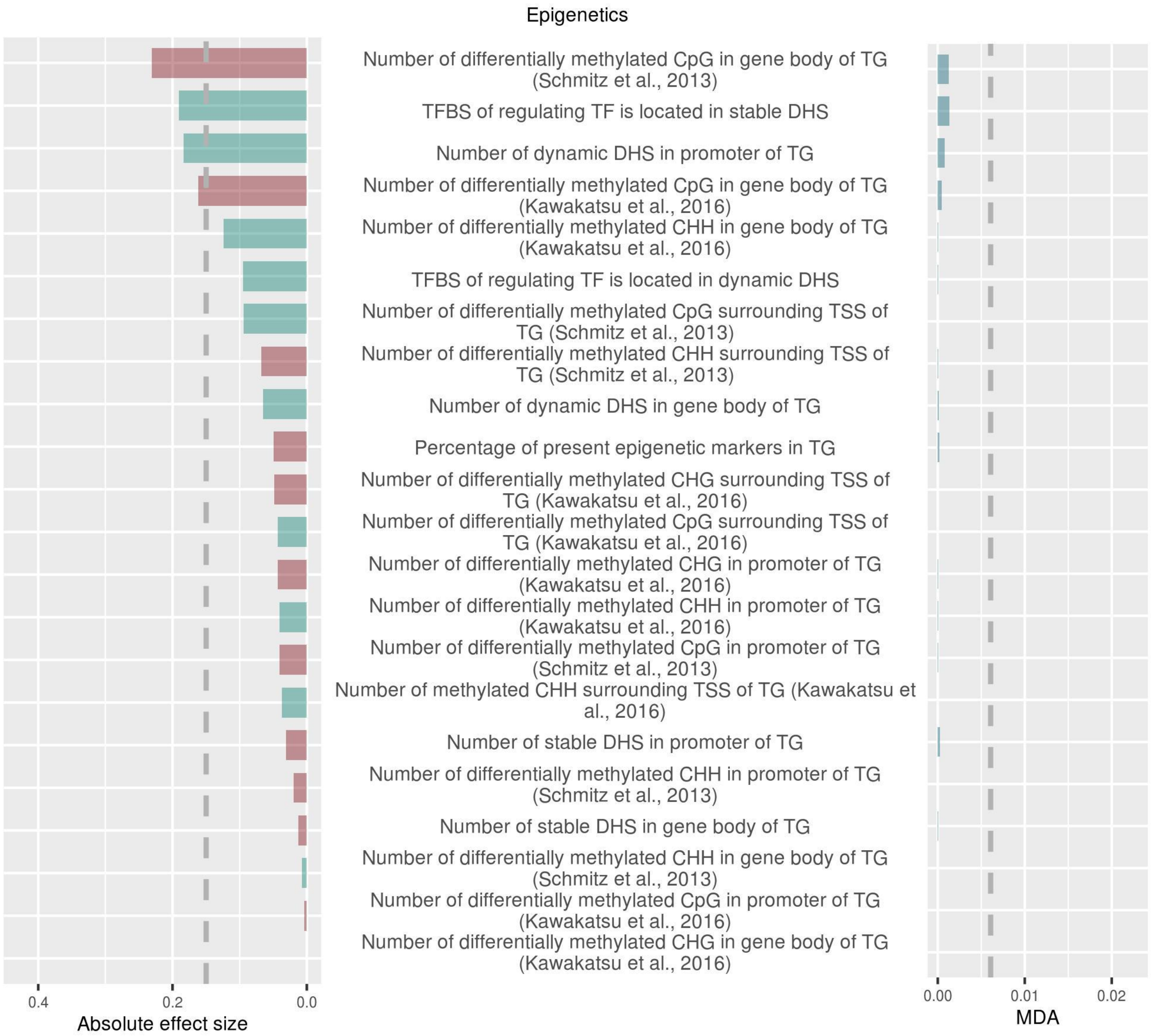

Supplementary Figure S6. Feature effect sizes and importance in the Random Forest classifier distinguishing between correlated and uncorrelated pairs for the feature class 'Epigenetics'. For additional information, see legend of Supplementary Figure S3.

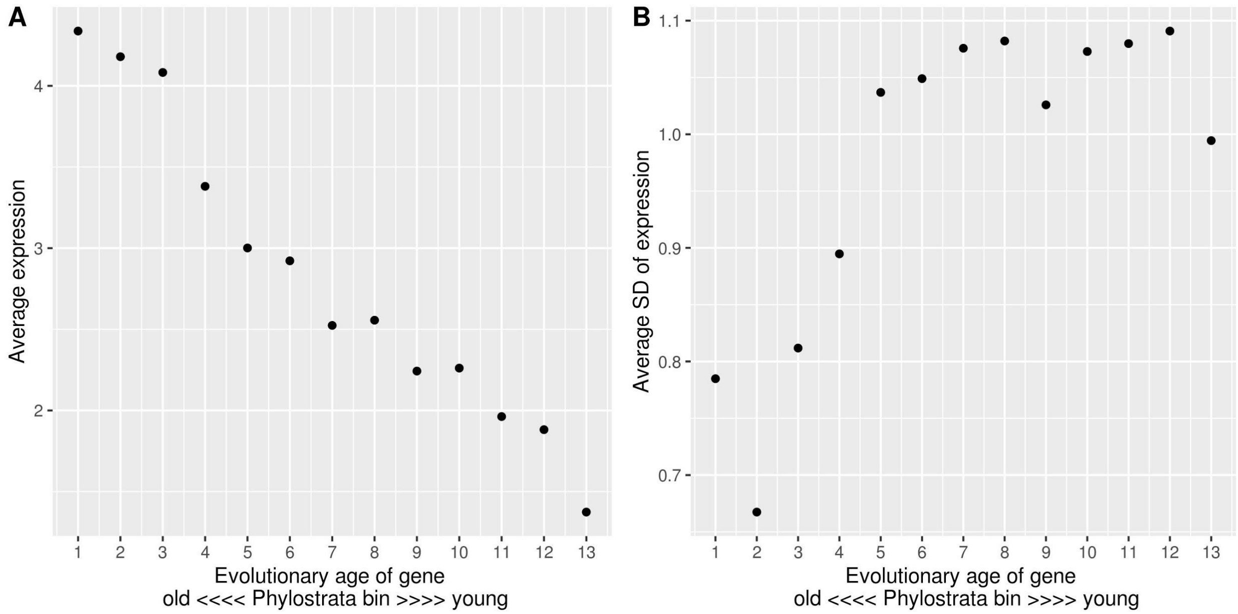

Supplementary Figure S7. Distribution of the expression average (A) and standard deviation (SD) (B) in relation to the evolutionary age of genes. Expression values were obtained from the 5,296 microarray hybridization set. For each gene in a selected phylostrata the average expression and SD was calculated and plotted as averaged of all gene in a respective age bin. Data on the evolutionary age from (1)

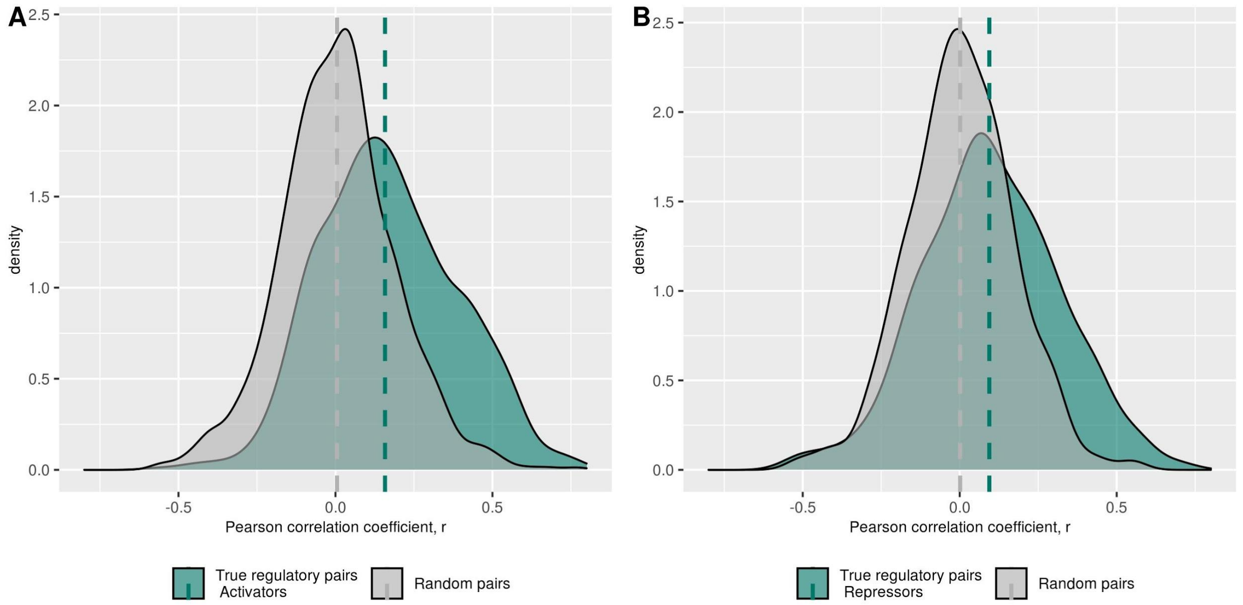

Supplementary Figure S8. Distribution of Pearson correlation coefficients of true TF-TG pairs expression of activators (A) and repressors (B). Plot was created for 998 TF-TG pairs of 262 activator TFs (A, green) and for 439 TF-TG pairs of 163 repressors (B, green). For comparison, set-size-matched distributions of the expression correlation were plotted (gray) for TF-random-TG pairs (random target genes were sampled from the set of TGs of other TFs present in the same set). Data from (2)

Supplementary Table S1. TF-families present in the set of active TFs. TF-transcription factor.

TF-family	Number of active TFs in TF-family	Total number of TFs in TF-family
AP2-EREBP	39	146
BBR/BPC	1	7
BES1	4	8
bHLH	2	136
bZIP	14	70
C2C2-Dof	5	36
C2C2-GATA	3	29
C2H2	3	99
C3H	1	68
CAMTA	1	6
E2F-DP	2	8
EIL	1	6
FAR1	1	17
HB	5	91
HSF	6	23
LOB	3	43
MYB	18	147
MYB-related	6	68
NAC	5	104
RWP-RK	1	14
S1Fa-like	1	3
TCP	5	24
Trihelix	6	23
WRKY	21	72

Supplementary Table S2. Set of Features considered as potential determinants of TF-TG correlation. Assignment of features to either transcription factor (TF), target genee (TG), or TF-TG pair is indicated in the “TF or TG or pair”-column. For each feature, mean values were calculated for all TF-TG pairs, which were considered as ‘Correlated’ and ‘Uncorrelated’, respectively. Effect size (Cohen’s D) and adjusted p-value of significance of difference based on Wilcoxon–Mann–Whitney test between ‘Correlated’ and ‘Uncorrelated’ pair sets. Data source or software used to collect or create selected features is listed in the column ‘source’. In cases of empty ‘source’-fields, feature was computed. PCD - “Plant Cistrome Database”. TF- transcription factor, TG -target gene, SD - standard deviation. (Table provided as separate Excel file.)

Supplementary Table S3. Set of all regulatory pairs and associated feature values collected for TFs, TGs, and regulatory pairs. Set of 157 active TFs. Provided are the original, individual features with their respective ranges and units, i.e. without scaling and clustering of features. Mapping of feature labels to names is given in the tab ‘Feature names legend’. (Table provided as separate Excel file.)

Supplementary Table S4. Importance of 112 clustered features determined by univariate testing and feature importance extraction from Random Forest models. Set of 112 clustered features for TF, TG and TF-TG pairs with median values for correlated pairs (Pearson correlation coefficient, $r > 0.4$) and uncorrelated pairs (absolute Pearson correlation coefficient, $|r| < 0.1$), effect size (Cohen’s D), FDR (Wilcoxon–Mann–Whitney test between ‘Correlated’ and ‘Uncorrelated’ pair sets adjusted with Benjamini-Hochberg correction) and Mean Decrease of Accuracy (MDA) calculated for Random Forest models (average over 20 cross-validation). In the category column abbreviations were used: ‘TF_TFBS_information’ = TF and TFBS information; ‘Post_PPI’ = Post-transcriptional, Post-translational and Protein-protein interactions (PPI); ‘Genomic’ = Genomic and genome annotation derived information; ‘Epigenetics’ = Epigenetics.

Supplementary Table S5. Enrichment of the GO process categories in old (PS1-PS3) and young (PS4-PS13) genes. Data on the evolutionary age from (1)

Old genes (PS1-PS3)				Young genes (PS4-PS13)		
**GO category**	**Fisher exact test, p_{FDR}**	**Odds ratio**		**GO category**	**Fisher exact test, p_{FDR}**	**Odds ratio**
**translation**	**5.74E-92**	**9.58**		**unknown biological processes**	**3.19E-263**	**3.61**
**generation of precursor metabolites and energy**	**3.74E-50**	**6.95**		**response to endogenous stimulus**	**9.58E-40**	**2.09**
**protein metabolic process**	**5.40E-48**	**2.31**		**signal transduction**	**7.77E-25**	**1.75**
**DNA metabolic process**	**1.72E-43**	**4.81**		**cell differentiation**	**4.54E-17**	**2.00**
**transport**	**1.40E-35**	**1.80**		**response to biotic stimulus**	**4.05E-14**	**1.64**
**photosynthesis**	**2.98E-33**	**6.94**		**secondary metabolic process**	**1.21E-11**	**2.20**
**cellular component organization**	**3.42E-29**	**1.60**		**response to chemical**	**1.64E-10**	**1.30**
**cell cycle**	**3.88E-24**	**2.63**		**cell-cell signaling**	**6.29E-07**	**14.51**
**lipid metabolic process**	**6.98E-14**	**1.68**		**growth**	**1.14E-06**	**1.53**
**catabolic process**	**1.64E-10**	**1.40**		**response to external stimulus**	**1.39E-06**	**1.32**
**embryo development**	**5.12E-10**	**1.80**		**carbohydrate metabolic process**	**1.08E-04**	**1.30**
**regulation of gene expression, epigenetic**	**1.14E-09**	**2.98**		**anatomical structure development**	**2.67E-04**	**1.17**
**cellular homeostasis**	**6.42E-08**	**1.93**		**flower development**	**3.62E-04**	**1.40**
**other cellular processes**	**6.29E-07**	**1.12**		**cell growth**	**3.62E-04**	**1.44**
**other metabolic processes**	**3.24E-06**	**1.12**		**abscission**	**7.82E-04**	**3.86**
**biosynthetic process**	**7.82E-04**	**1.12**		cellular protein modification process	1.62E-03	1.15
regulation of molecular function	6.46E-03	1.38		fruit ripening	1.71E-03	0
post-embryonic development	4.42E-02	1.12		tropism	3.24E-03	1.88
response to light stimulus	6.70E-02	1.14		multicellular organism	5.67E-03	1.12

				development		
nucleobase-containing compound metabolic process	1.31E-01	1.05		response to abiotic stimulus	4.01E-02	1.11
circadian rhythm	1.67E-01	1.24		response to stress	4.42E-02	1.07
DNA binding	3.15E-01	0		pollination	7.05E-02	1.24
protein binding	3.22E-01	1.29		other biological processes	1.25E-01	1.11
reproduction	4.01E-01	1.02		catalytic activity	2.42E-01	4.00
nucleic acid binding	5.00E-01	0		other binding	3.77E-01	3.00
transcription regulator activity	5.00E-01	0		cell death	4.74E-01	1.05
RNA binding	5.00E-01	0		transferase activity	5.00E-01	0
other molecular functions	5.00E-01	0		DNA-binding transcription factor activity	5.00E-01	0
				transporter activity	5.00E-01	2.00
				cell communication	5.00E-01	1.01

Supplementary Table S6. List of TF-families enriched in positive and negative correlated and uncorrelated TF-TG pairs for the set of active TFs. Significance was calculated based on the Fisher exact test for enrichment in the set of correlated pairs (|r|>0.4) vs. uncorrelated (|r|<0.1) pairs with FDR correction for multiple testing. Odds ratios represent relative enrichment of counts in correlated vs. uncorrelated pairs. Note that counts entering the enrichment statistic and computed p-values were based on TF-TG pairs, such that every TF entered the statistic based on all its TGs.

TF-families enriched in positively and negatively correlated TF-TG pairs		
**TF-family**	**Fisher exact test, p_{FDR}**	**Odds ratio**
WRKY	0.00E+00	3.30
MYB	1.06E-28	1.57
TCP	2.83E-20	2.73
HB	9.03E-10	1.51
MYB-related	5.68E-02	1.10
E2F-DP	8.84E-02	1.34
NAC	8.96E-02	1.13
EIL	4.04E-01	1.20
**TF-families enriched in uncorrelated TF-TG pairs**		
AP2-EREBP	1.07E-198	0.22
C2C2-Dof	1.28E-48	0.34
BBR/BPC	1.41E-39	0.04
C2C2-GATA	8.80E-37	0.00
C3H	1.65E-24	0.11
bZIP	1.67E-23	0.61
BES1	3.02E-23	0.31
S1Fa-like	3.07E-14	0.00
CAMTA	4.71E-14	0.05
HSF	2.24E-10	0.42
bHLH	2.11E-09	0.07
LOB	4.31E-06	0.46
Trihelix	8.57E-05	0.60
FAR1	1.21E-02	0.28
RWP-RK	3.19E-01	0.61
C2H2	4.61E-01	0.98

References

1. Quint,M., Drost,H.-G., Gabel,A., Ullrich,K.K., Bönn,M. and Grosse,I. (2012) A transcriptomic hourglass in plant embryogenesis. *Nature*, **490**, 98–101.
2. Jin,J., He,K., Tang,X., Li,Z., Lv,L., Zhao,Y., Luo,J. and Gao,G. (2015) An Arabidopsis Transcriptional Regulatory Map Reveals Distinct Functional and Evolutionary Features of Novel Transcription Factors. *Mol. Biol. Evol.*, **32**, 1767–1773.
